# Quality and capacity indicators for hospitalized pediatric oncology patients with critical illness: A modified delphi consensus

**DOI:** 10.1002/cam4.3351

**Published:** 2020-08-10

**Authors:** Anita V. Arias, Marcela Garza, Srinivas Murthy, Adolfo Cardenas, Franco Diaz, Erika Montalvo, Katie R. Nielsen, Teresa Kortz, Rana Sharara‐Chami, Paola Friedrich, Jennifer McArthur, Asya Agulnik

**Affiliations:** ^1^ Division of Pediatric Critical Care University of Tennessee Health Science Center Memphis TN USA; ^2^ Department of Global Pediatric Medicine St. Jude Children’s Research Hospital Memphis TN USA; ^3^ Department of Pediatrics University of British Columbia Vancouver BC Canada; ^4^ Hospital Infantil Teletón de Oncología (HITO) Querétaro México; ^5^ Facultad de Medicina Clínica Alemana Universidad del Desarrollo Santiago Chile; ^6^ Pediatric Critical Care Unit SOLCA Quito Quito Ecuador; ^7^ Division of Pediatric Critical Care University of Washington Seattle WA USA; ^8^ Department of Global Health University of Washington Seattle WA USA; ^9^ Division of Pediatric Critical Care University of California San Francisco San Francisco CA USA; ^10^ Department of Pediatric and Adolescent Medicine American University of Beirut Medical Center Beirut Lebanon; ^11^ Division of Pediatric Critical Care St. Jude Children’s Research Hospital Memphis TN USA

**Keywords:** clinical cancer research, pediatric cancer, translational research

## Abstract

**Background:**

Hospitalized pediatric hematology‐oncology (PHO) patients are at high risk for critical illness, especially in resource‐limited settings. Unfortunately, there are no established quality indicators to guide institutional improvement for these patients. The objective of this study was to identify quality indicators to include in PROACTIVE (**P**ediat**R**ic **O**ncology c**A**pa**C**ity assessment **T**ool for **I**ntensi**V**e car**E**), an assessment tool to evaluate the capacity and quality of pediatric critical care services offered to PHO patients.

**Methods:**

A comprehensive literature review identified relevant indicators in the areas of structure, performance, and outcomes. An international focus group sorted potential indicators using the framework of domains and subdomains. A modified, three‐round Delphi was conducted among 36 international experts with diverse experience in PHO and critical care in high‐resource and resource‐limited settings. Quality indicators were ranked on relevance and actionability via electronically distributed surveys.

**Results:**

PROACTIVE contains 119 indicators among eight domains and 22 subdomains, with high‐median importance (≥7) in both relevance and actionability, and ≥80% evaluator agreement. The top five indicators were: (a) A designated PICU area; (b) Availability of a pediatric intensivist; (c) A PHO physician as part of the primary team caring for critically ill PHO patients; (d) Trained nursing staff in pediatric critical care; and (e) Timely PICU transfer of hospitalized PHO patients requiring escalation of care.

**Conclusions:**

PROACTIVE is a consensus‐derived tool to assess the capacity and quality of pediatric onco‐critical care in resource‐limited settings. Future endeavors include validation of PROACTIVE by correlating the proposed indicators to clinical outcomes and its implementation to identify service delivery gaps amenable to improvement.

## Graphical abstract

In this study, an international expert panel with diverse experience in oncology and critical care in high and low resource settings, identified and selected the most relevant capacity and quality indicators to be included in a tool to measure service delivery gaps for critically ill pediatric hematology‐oncology patients in resource‐limited settings.

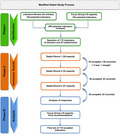

## 1 INTRODUCTION

Survival rates for children with cancer have dramatically increased during the past decades to over 80% in high‐resource settings^1^, ^2^, ^3^ in part due to chemotherapy and radiotherapy regimens, bone marrow transplantation, effective and aggressive surgeries, and the comprehensive supportive care provided to these patients. Unfortunately, more than 80% of pediatric cancers occur in low‐ and middle‐income countries (LMICs), where survival rates are reported between 10% and 50%.^3^, ^4^

Hospitalized pediatric hematology‐oncology (PHO) patients are at high‐risk for clinical deterioration and mortality, with up to 40% of all pediatric oncology patients requiring admission to the PICU at some point in their disease course.^5^, ^6^, ^7^, ^8^ A meta‐analysis by Wösten‐van Asperen et al. showed that the overall PICU mortality for children with cancer in high‐resource settings was 27.8%, with higher mortality in patients requiring mechanical ventilation, inotropic support, renal replacement therapy, and those treated outside a pediatric intensive care unit (PICU).^5^, ^6^, ^7^, ^9^

In recent years, initiatives to improve global childhood cancer survival have gained awareness through the World Health Assembly Cancer Resolution (May 2017) and the World Health Organization Global Initiative for Childhood Cancer (September 2018).^10^, ^11^ Under this perspective, there is also a growing need to address critical illness as part of the global programs to strengthen capacities, reduce mortality, and improve outcomes for cancer patients. Despite the need to guarantee high‐quality care to critically ill PHO patients, multiple factors affect the ability to provide adequate critical care in resource‐limited settings, including resource scarcity, limited staff and provider awareness, and inadequate access to pediatric intensive care,^12^ resulting in poor patient outcomes and high inpatient mortality.

To design successful improvement initiatives in the care of critically ill PHO patients, it is fundamental for hospitals to objectively measure and assess baseline capacity and quality of care provided. Quality indicators are increasingly being used to measure and improve the quality of healthcare and enable evidence‐based planning, management, and policy development.^13^ Quality metrics for benchmarking and tracking improvements in safety and quality of care for critically ill pediatric patients have previously been described.^14^, ^15^ However, valid indicators to assess the quality of care processes and performance for critically ill PHO patients are lacking, hence clinicians have no means to identify areas for targeted interventions.

The **P**ediatric **O**ncology **F**acility **I**ntegrated **L**ocal **E**valuation (PrOFILE) Tool was developed to help institutions identify improvement strategies and optimal care delivery for pediatric oncology patients.^16^ However, this tool does not comprehensively address characteristics of care for PHO patients with critical illness. The goal of this work was to identify the most relevant capacity and quality indicators for critically ill PHO patients with underlying malignant disease (including leukemias, lymphomas, solid tumors and posthematopoietic cell transplant patients) in resource‐limited settings using a modified Delphi consensus approach^17^, ^18^, ^19^ and to develop the **P**ediat**R**ic **O**ncology c**A**pa**C**ity assessment **T**ool for **I**ntensi**V**e car**E (**PROACTIVE).

## 2 METHODS

This study received an IRB exemption by St. Jude Children's Research Hospital and the University of Tennessee Health Science Center (Memphis, TN) and was performed in stages as follows:

### 2.1 Search strategy

A preliminary list of potential quality indicators was created by a comprehensive electronic literature review conducted from May to June 2018, through the PubMed/Medline, Scopus and Web of Science databases, using the following key words: “pediatric intensive care unit”, “pediatric critical care”, “pediatric oncology”, “pediatric cancer”, “quality markers”, “quality indicators”, “capacity”, “quality of care”, “quality improvement”, “standards of care”, “performance measures”, “performance metrics”, “low‐income countries”, and “limited resources”. Search was restricted by language (English) and date of publication (January 1, 1998‐April 30, 2018). Complete details of search strategy are provided in Table S1.

Retrieved titles, citations and abstracts were reviewed by one of the authors (AV.A.) who identified potential eligible articles. The full‐text versions of these studies were then reviewed by two authors (AV.A., AA) to identify quality indicators for critically ill PHO patients in resource‐limited settings. We excluded studies where outcomes were not focused on pediatric critically ill patients or that were not conducted in an ICU environment, as well as studies not available in full‐text (comments, editorials, letters, review articles, or conference papers). References and details of the included studies for the development of quality indicators can be found in Table S2.^9^, ^14^, ^15^, ^20^, ^21^, ^22^, ^23^, ^24^, ^25^, ^26^, ^27^, ^28^, ^29^, ^30^, ^31^, ^32^, ^33^, ^34^, ^35^, ^36^, ^37^, ^38^, ^39^, ^40^

### 2.2 Selection of quality indicators

An international expert focus group (n = 10) was carefully selected to ensure disciplinary representation (pediatric oncologists and intensivists), research expertise and regional representation (Table 1), with eight members of the focus group either primarily working in or having extensive experience in resource‐limited settings as part of their academic work. The focus group reviewed and sorted the most relevant indicators using the framework of domains and subdomains previously developed for the **P**ediatric **O**ncology **F**acility **I**ntegrated **L**ocal **E**valuation (PrOFILE) tool.^16^ Details provided in Table S3. Selected indicators were arranged into domains and subdomains and adapted as an online survey for evaluation by an international panel of experts (n = 36) to determine their relevance and actionability.

**TABLE 1:** Demographics and characteristics of the expert focus group

Category	Focus Group (n,%)
Gender	
Female	7 (70%)
Male	3 (30%)
Position in organization	
Pediatric Intensivist	6 (60%)
Pediatric Oncologist	1 (10%)
Pediatric Onco‐Critical Care	3 (30%)
Years of Experience	
<5 y	5 (50%)
5‐10 y	2 (20%)
>10 y	3 (30%)
Country of Primary Practice	
United States of America	5 (50%)
Canada	1 (10%)
Mexico	1 (10%)
Ecuador	1 (10%)
Chile	1 (10%)
Lebanon	1 (10%)
PICU Location of Primary Practice	
High‐Income Country (HIC)^a^	7 (70%)
Middle‐Income Country (MIC)^b^	3 (30%)
Actively Working in RLS (Clinical, Research)	
Yes	8 (80%)
No	2 (20%)

Abbreviations: RLS, Resource‐Limited Settings.

^a,b^ Based on the World Bank classification: we classified as High‐income countries (HIC) those with a gross national income per capital (GNI) of ≥ US$12 375, as Middle‐income countries (MIC) those with a GNI of US$1026‐US$12 375 (includes lower‐ and upper‐middle‐income countries) and Low‐income countries (LIC) those with a GNI ≤ US$1026.

### 2.3 Participants

International experts were identified using the St. Jude Global's network of pediatric oncology centers, prior relevant publications, and suggestions from the focus group. Thirty‐six experts accepted the electronic invitation to participate in the study, including the original focus group. Commitment to contribute to all rounds was requested when agreeing to participate in our study. Demographics of the expert panel and participation in the three rounds are presented in Table 2. Participating experts had diverse experience in pediatric oncology and critical care in high‐resource and resource‐limited settings, with greater than 80% of the panelist having experience working in settings with resource limitations. Panelists came from 18 countries (Table S4) of diverse income levels according to the World Bank classification.^41^ We aimed to retain at least 75% participation, which was achieved by the end of our modified Delphi process.

**TABLE 2:** Demographics and characteristics of expert panel

Category		Round 1 (n; %)	Round 2 (n; %)	Round 3 (n; %)
Total	Participants	36 (100%)	32 (88.0%)	27 (75.0%)
Gender	Female	18 (50.0%)	16 (50.0%)	13 (48.1%)
	Males	18 (50.0%)	16 (50.0%)	14 (51.9%)
Age	30‐40 y	16 (44.4%)	15 (46.9%)	13 (48.1%)
	41‐50 y	11 (30.6%)	9 (28.1%)	9 (33.3%)
	51‐60 y	7 (19.4%)	6 (18.8%)	4 (14.8%)
	>61 y	2 (5.6%)	2 (6.3%)	1 (3.7%)
Specialty	Pediatric Intensivists	28 (77.8%)	26 (81.3%)	22 (81.5%)
	Pediatric Oncologists	6 (16.7%)	5 (15.6%)	5 (18.5%)
	Nurses	2 (5.6%)	1 (3.1%)	0 (0%)
Years of Experience	<5 y	10 (27.8%)	10 (31.3%)	9 (33.3%)
	5‐10 y	8 (22.2%)	7 (21.9%)	5 (18.5%)
	>10 y	18 (50.0%)	15 (46.9%)	13 (48.1%)
Region^a^	North America (USA & Canada)	10 (27.8%)	10 (31.3%)	9 (33.3%)
	North America (Mexico)	6 (16.7%)	5 (15.6%)	2 (7.4%)
	Central America ‐ Caribbean	6 (16.7%)	5 (15.6%)	4 (14.8%)
	South America	5 (13.9%)	5 (15.6%)	5 (18.5%)
	Europe	3 (8.3%)	3 (9.4%)	3 (11.1%)
	Asia	4 (11.1%)	3 (9.4%)	4 (14.8%)
	Africa	2 (5.6%)	1 (3.1%)	0 (0%)
Country Income Level^b^	HIC	16 (44.4%)	14 (43.8%)	14 (51.9%)
	MIC (lower and upper MIC)	17 (47.2%)	16 (50.0%)	11 (40.7%)
	LIC	3 (8.3%)	2 (6.3%)	2 (7.4%)
Actively working in RLS (Clinical and Research)	Yes	30 (83.3%)	27 (84.4%)	22 (81.5%)
	No	6 (16.7%)	5 (15.6%)	5 (18.5%)

Abbreviations: HIC, High‐Income Countries; LIC, Low‐Income Countries; MIC, Middle‐Income Countries; RLS, Resource‐Limited Settings.

^a^ Table with the participating countries can be found onTable S4

^b^ Based on the World Bank classification: we classified as High‐income countries (HIC) those with a gross national income per capital (GNI) of ≥US$12,375, as Middle‐income countries (MIC) those with a GNI of US$1,026‐US$12,375 (includes lower‐ and upper‐middle‐income countries) and Low‐income countries (LIC) those with a GNI ≤US$1026.

### 2.4 Consensus rounds

To achieve consensus in a final set of capacity and quality indicators, a three‐round modified Delphi survey was executed among a group of 36 international experts over a period of 6 months followed by a Focus Group meeting (n = 10) to ensure capture of the most important indicators. All surveys were designed online using the secure online survey software COMET Initiative Delphi Manager.^42^

During all three rounds, experts were asked to rank each indicator according to its relevance (the indicator captures key aspects in the clinical process) and actionability (the indicator can be acted upon to improve patient care), using a Likert‐scale ranging from 1 (not important) to 9 (very important). Experts could select “unable to score” if they felt they lacked sufficient experience or knowledge to rate an indicator. Responses were collected over a 3‐4‐week period. Non‐responders received up to two reminders prior to the date of closure for each of the rounds.

### 2.5 Consensus criteria

Criteria for acceptance and rejection of indicators were determined a priori. An indicator was accepted into the final set of candidate indicators when it scored in the upper tertile (7‐9) on both relevance and actionability and reached ≥ 80% agreeability among the experts. An indicator was rejected when it scored in the lowest tertile (1‐3). An indicator was classified as uncertain if it scored 4‐6 and had significant disagreement among experts, thus advancing to the next rounds.^17^

### 2.6 Top indicators

At the end of the consensus rounds, the focus group selected the top indicators in each domain. The focus group opinion was defined by calculating the percentage of responses for each indicator and agreed on by absolute majority.

### 2.7 Data analysis

Results from all rounds were analyzed using Excel 2016 and SPSS to calculate median importance (MI) and percent agreeability among the experts. The “unable to score” responses were excluded from calculations.

## 3 RESULTS

**FIGURE 1:**
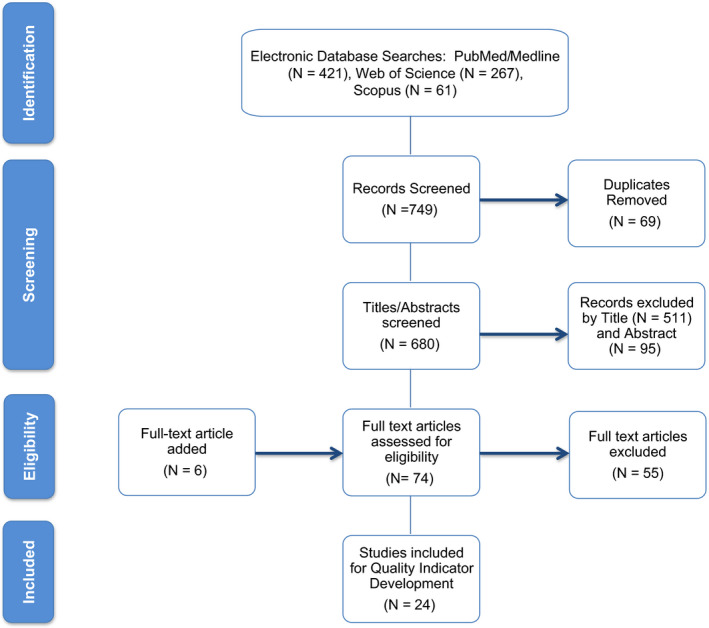
Flow diagram of studies selection. The PRISMA flow diagram details the process of article identification and selection for inclusion. The initial database search resulted in 749 records; after duplicates removed, 680 abstracts were screened. This process left 74 records to assess for eligibility by screening the full‐text articles. An additional six records were identified from other sources. Twenty‐four articles were finally included for the development of quality indicators. PRISMA = preferred reporting items for systematic reviews and meta‐analyses

**FIGURE 2:**
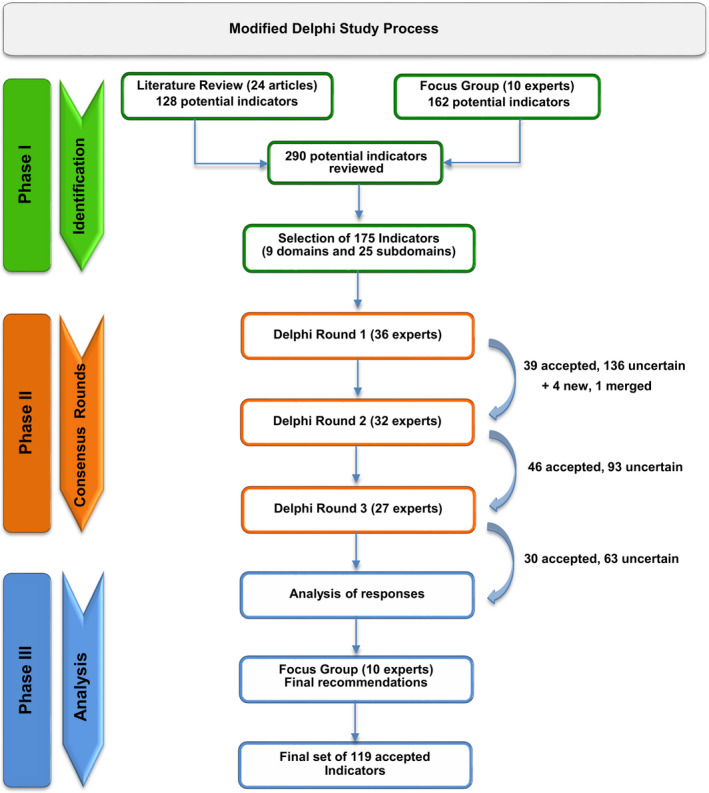
Modified delphi study algorithm. An initial set of 290 possible quality indicators were identified. The focus group narrowed the list to 175 potential indicators to be included in the consensus rounds. Only indicators with high median importance (score of 7‐9) in both relevance (captures key aspects in the clinical process) and actionability (can be acted upon to improve patient care) and ≥80% evaluator agreement were selected as part of the final set of capacity and quality indicators

The literature search generated a list of 749 possible articles for review, of which 24 publications met inclusion criteria to be reviewed for identification of possible capacity and quality indicators for critically ill pediatric oncology patients (Figure 1). An initial set of 290 possible indicators were identified. The focus group narrowed the list to 175 potential indicators among nine domains and 25 subdomains. These indicators were included in the Modified Delphi Consensus process described in Figure 2.

### 3.1 Consensus rounds

**FIGURE 3:**
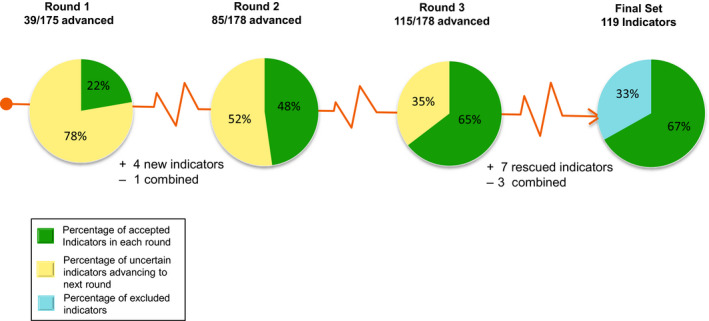
Percentage of quality indicators that achieved consensus. The final PROACTIVE tool contains 119 capacity and quality indicators. The experts added four new indicators and merged two indicators after round one, totaling 178 indicators for ranking on a scale of 1‐9. A total of 115 indicators achieved consensus after three consensus rounds. The focus group rescued seven indicators and combined three indicators, totaling 119 indicators that were finally accepted into the PROACTIVE tool

Of the 175 potential indicators presented to the panel experts in round one, 39 indicators were accepted into the final tool. During this round, the expert panel refined wording of the capacity and quality indicators, added four new indicators and merged two similar indicators (Figure 3). In rounds two and three, 46 and 30 indicators were accepted, respectively. No indicator was rated as unimportant during the three consensus rounds. The proportion of “unable to score” responses were 0.01%, 0.04%, and 0.01%, respectively, during each round.

After three rounds of consensus, 115 indicators were accepted as part of the final set of quality indicators, 55 were excluded as they attained scores 4‐6, and 18 were classified as uncertain as they reached scores in the upper tertile (7‐9) but did not obtain ≥80% expert agreeability for inclusion into the final PROACTIVE tool (Figure 3). The focus group was then asked to review the 115 accepted indicators and the 18 uncertain indicators. The focus group reached consensus on inclusion of 7/18 uncertain indicators and merged similar indicators for a total of 119 indicators among eight domains and 22 subdomains to be included in the PROACTIVE tool (See Table S5 and S6).

The focus group also selected the top five most important indicators along with the most important indicators in each domain, totaling 11 indicators identified as priority in the care for children with cancer who develop critical illness (Table 3). The overall top five most important indicators selected by absolute majority were: (a) A designated PICU area; (b) Availability of a pediatric critical care physician; (c) Inclusion of a hematology‐oncology physician as part of the primary team caring for critically ill PHO patients; (d) Trained nursing staff in pediatric critical care; and (e) Timely PICU transfer of hospitalized PHO patients who require escalation of care.

**TABLE 3:** Top indicators per domains: the highest rated capacity and quality indicators by the focus group

Domains	Top indicators
National context	Presence of a national, publicly funded healthcare program (endorsed by the Ministry of Health) that provides coverage for pediatric critical illness.
Facility and local context	Presence of a designated PICU area (designated area within a hospital), separated from other inpatient locations (eg. general ward).
Personnel	Availability of a pediatric critical care physician as part of the primary medical team responsible for the care of critically ill PHO patients. Availability of a pediatric hematology‐oncology physician as part of the primary medical team responsible for the care of critically ill PHO patients. Availability of nursing staff trained in pediatric critical care as part of the primary medical team responsible for the care of critically ill PHO patients.
Service capacity	Timely transfer (within 4 hr.) of hospitalized PHO patients who require escalation of care to the PICU from other hospital units (eg. general floor).
Service integration	Daily multidisciplinary patient care rounds led by a pediatric critical care physician for hospitalized critically ill PHO patients.
Supportive services	Frequency of inadequate pediatric critical care nurse staffing affecting the management of critically ill PHO patients.
Medication and equipment	Consistent access to first line antibiotics for critically ill PHO patients presenting with fever and neutropenia. Consistent access to monitoring equipment with alarm systems indicating critical values and continuous monitoring capabilities at each bedside of critically ill PHO patients.
Outcomes	Presence of a patient data registry that includes mortality of hospitalized PHO/BMT patients in the PICU/IMCU.

Abbreviations*:* BMT, bone marrow transplant; IMCU, intermediate medical care unit; PHO, pediatric hematology‐oncology patient; PICU, pediatric intensive care unit.

## 4 DISCUSSION

In this study, we described the development of the PROACTIVE tool, identifying and selecting 119 evidenced and consensus‐based quality indicators to measure capacity gaps for critically ill PHO patients in resource‐limited settings among 8 fundamental domains: (a) National Context, (b) Facility and Local Context, (c) Personnel, (d) Service Capacity, (e) Service Integration, (f) Supportive Services, (g) Medication and Equipment, and (h) Outcomes.

Several studies have previously attempted to define ideal quality indicators in pediatric critical care, including indicators such as mortality rate, hospital length of stay, and safety metrics to eliminate errors and risk of injuries for these patients^14^, ^15^; however, there remains a lack of gold standards in this field. To our knowledge, this is the first study to identify capacity and quality indicators for critically ill PHO patients. These indicators can be used to map resources and health services available in resource‐limited settings, including hospitals in high‐income countries with resource limitations. The final PROACTIVE tool will be administered as an electronic survey divided in eight modules, one per each domain, and will contain questions based on the 119 selected indicators with defined answers in the Likert, numerical, and Boolean scale to facilitate scoring of results (see Table S6 for a preliminary version of the tool). The collected information will be presented in tables and graphs to help stakeholders understand the relative strengths and limitations of their pediatric onco‐critical care services. Implementation of the tool, with relevant metrics and analysis, will be supported by St. Jude Global as part of their mission to improve survival of children with cancer through the sharing of knowledge, technology, and organizational skills.^43^ Hence, PROACTIVE will act as a diagnostic tool that will create a baseline database of service capacities and resources for pediatric onco‐critical care in participating centers, while monitoring and highlighting areas in need of further in‐depth assessment. Additionally, it will allow stakeholders to collaboratively prioritize among multiple potential quality improvement interventions at their individual facility and permit insightful benchmarking at the local and regional level. By repeating the PROACTIVE tool, centers will also be able to track their institution's performance and progress over time.

As part of the consensus process, we identified the top five most important indicators to highlight high‐priority issues for managing critical illness in PHO patients in resource‐limited settings. These indicators were chosen by the focus group to emphasize that the presence of a designated PICU area, the availability of trained nurses and specialists, as well as early PICU admission for patients requiring escalation of care are essential characteristics of high‐quality pediatric onco‐critical care and can be achieved through prioritization and allocation of available resources. While establishing intensive care access with availability of trained pediatric critical care physicians and nurses might be challenging in resource‐limited settings, these services provide early and essential care for critically ill children, reducing their overall morbidity and mortality.^23^, ^44^, ^45^ Appropriate PICU admission for critically ill PHO patients is also challenging in settings with limited PICU beds; however, timely transfer of PHO patients requiring escalation of care is an equally important and cost effective way to improve outcomes.^4^, ^21^, ^26^, ^46^ In critically ill adult patients with cancer delayed ICU transfers (≥4 hours) is associated to increased morbidity and mortality.^47^, ^48^ As similar concepts apply in pediatrics, early PICU admission can reduce resource utilization by allowing for early initiation of therapy and reducing progression of organ dysfunction and death. Facilities caring for these children with cancer should implement systems to facilitate timely transfer of critically ill PHO patients to the PICU, including the use of validated tools such as PEWS^21^ to detect patients at risk of acute deterioration and algorithms with PICU admission criteria to expedite transfer of these patients.^6^, ^46^ Such interventions help emphasize that most critically ill PHO children would benefit from PICU services and should be considered eligible to receive maximal therapy. Finally, inclusion of a hematology‐oncology physician as part of the primary team caring for critically ill PHO patients is of paramount importance to improve outcomes as their expertise is essential to the optimization of diagnostic and treatment practices. A multidisciplinary approach with daily meetings between the intensivist and the oncologist can facilitate goals of care discussion and treatment strategies, enhance a culture of continuous communication and ensure continuity of high‐quality care for these children.^6^ These high‐priority topics align with the recent proposed research priorities by the ESPNIC and POKER groups in Europe to coordinate and improve onco‐critical care for children with cancer at an international level.^49^

In our study, we included indicators that do not specifically refer to onco‐critical care, but represent general supportive and critical care quality indicators. This was a deliberate decision by the focus group, as critical care for children with cancer does not happen in a vacuum within a hospital, and the general quality of critical care services impacts the quality of services delivered to children with cancer. Similarly, improvements for critical care services for pediatric oncology patients in a hospital would likely lead to improvements for all pediatric patients.

This study has several limitations. There is a lack of literature supporting the validity and reliability to measure outcomes of many of the proposed indicators. However, we utilized a modified Delphi consensus method, which has been used successfully in the development of core outcomes and quality indicators in health‐related research.^18^, ^19^ During the literature search, a single reviewer screened eligible studies. While the Cochrane Collaboration recommends the use of two reviewers, they also indicate that a single screening approach may be adequate and that a second reviewer in the full‐text stage may be sufficient.^50^ As we presented an initial large set of identified indicators to the panel of experts and convened a focus group to further select final indicators, we believe this methodology decreased the risk of missing potentially relevant indicators and minimized bias. Although the expert panel was geographical diverse, the majority practice in middle‐income countries, which may limit the applicability of the selected indicators in low‐income countries. PROACTIVE, however, is geared toward centers that manage children with cancer in resource‐limited settings, regardless of the country's income level, and our experts had diverse experience working in these settings. For these reasons, we are confident that the developed tool offers appropriate guidance for the majority hospitals with resource limitations managing critically ill pediatric oncology patients.

In our study, we had difficulty narrowing potential indicators given that no indicators were eliminated during the consensus rounds. Although we used rigid selection criteria, we finally accepted a relatively large number of indicators deemed applicable to quality improvement efforts. While the broad number of indicators may appear burdensome and may make it difficult to prioritize interventions in resource‐limited settings, the included indicators were selected by a geographical diverse and multidisciplinary expert panel, suggesting relevance across many regions and allowing for a more comprehensive and inclusive assessment tool. The categorization of indicators by domains and subdomains may also aid in the improvement efforts across multiple levels at hospitals caring for critically ill PHO patients.

As part of our consensus process, no quality indicators in the Finance domain reached the required ≥ 80% evaluator agreement despite scoring in the upper tertile (7‐9) on both relevance and actionability. As a result, this was the only domain eliminated by the panel of expert. One cost‐related indicator in the National Context domain (presence of a national publicly funded healthcare program), however, was selected by the expert panel and highlighted by the focus group as a high priority issue in resource‐limited settings. The presence of a national publicly funded healthcare program would likely address the indicators not accepted in the Finance domain. Similarly, many accepted indicators focus on practices, such as early identification of critical illness in PHO patients, expected to lead to better utilization already scarce resources and improved cost effectiveness. Nevertheless, further studies are needed to evaluate how implementation of universal health financing systems and out‐of‐pocket health expenditures affect the outcomes of cancer patients in resource‐limited settings.

Despite these limitations, the strengths of our study include the use of a comprehensive literature review, focus group meetings to allow robust discussion, and inclusion of a large number of indicators selected by experts from different disciplines and regions. We also aimed to minimize participants’ attrition by ensuring our survey was technically unchallenging, provided clear completion instructions, and analyzed the data quickly in between rounds to keep the experts engaged in the process. Some experts may have dropped out of the study due to survey fatigue given the length of our questionnaire or due to competing priorities with other work. Nevertheless, we obtained 75% or greater panelists participation in each round of the Delphi, which is sufficient to minimize response bias (recommended > 70% response rate).^18^

Our work has clear relevance for the multidisciplinary teams caring for pediatric oncology patients, and it is an important step toward improving the quality of care for critically ill pediatric oncology patients.

PROACTIVE aims to provide a comprehensive, modular, and guided institutional self‐assessment and will allow multiple stakeholders identify and prioritize solutions adapted to their institution's need. Monitoring the indicator data will help institutions target specific quality improvement initiatives and by repeating the tool they will be able to track their performance and measure whether quality of care is improving over time.

Future endeavors include piloting the electronic PROACTIVE tool for implementation, correlating the proposed indicators to clinical outcomes for validation, and testing our hypothesis that the use of PROACTIVE can help institutions identify service delivery gaps amenable to improvement. We anticipate that after implementation of our evidence‐based tool, clinicians and organizations would be able to understand their current state, identify specific service delivery gaps, compare their performance over time, and benchmark themselves to their country and region, strengthening their inpatient services and the overall survival of children with cancer worldwide.

## 5 CONCLUSION

Hospitalized PHO patients have frequent life‐threatening complications that may require intensive care. Pediatric critical care is an essential service for this high‐risk population, especially in LMICs where clinicians are challenged to manage these patients with limited resources. This study describes the development of PROACTIVE, an evidence‐ and consensus‐derived tool to assess capacity and quality of care for critically ill PHO patients in resource‐limited settings. By using this tool, we believe that institutions and practitioners caring for acutely ill PHO patients can potentially track their performance and develop targeted interventions to improve critical care capacity and services delivered. Furthermore, our approach to identify the most relevant quality indicators for pediatric oncology critical care may be useful to others attempting to better understand the heterogeneity of critical care resources available for pediatric oncology patients in their settings and achieve the overall goal of improving the survival of children with cancer worldwide.

## CONFLICT OF INTEREST

The authors have no conflict of interests to disclose.

## AUTHOR CONTRIBUTIONS

Conceptualization: A. Arias, A. Agulnik; Methodology: A. Arias, A. Agulnik, P. Friedrich; Software: A. Arias; Data Curation: A. Arias, A. Agulnik, M. Garza; Formal Analysis: A. Arias, A. Agulnik, M. Garza; Investigation: A. Arias, A. Agulnik, A. Cardenas, F. Diaz, P. Friedrich, M. Garza, J. McArthur, S. Murthy, E. Montalvo, K. R. Nielsen, T. Kortz, R. Sharara‐Chami; Resources: A. Arias, A. Agulnik; Validation: A. Arias, A. Agulnik; Visualization: A. Arias, A. Agulnik; Project Administration: A. Arias, A. Agulnik; Supervision: A. Agulnik, P. Friedrich, J. McArthur; Funding Acquisition: A. Arias, A. Agulnik; Writing‐original draft: A. Arias; Writing‐review and editing: A. Arias, A. Agulnik, A. Cardenas, F. Diaz, P. Friedrich, M. Garza, J. McArthur, S. Murthy, E. Montalvo, K. R. Nielsen, T. Kortz, R. Sharara‐Chami. All authors contributed and approved the final manuscript as submitted and agree to be accountable for all aspects of the work.

## Supporting information

Supplementary Material

## Data Availability

All relevant data that support the findings of this study are available within the paper and the supplementary material of this article.
