# Supplementary material for: Quality and capacity indicators for hospitalized pediatric oncology patients with critical illness: A modified delphi consensus
Source: Cancer Med. 2020 Aug 10;9(19):6984–95. doi: 10.1002/cam4.3351 (PMC7541142; doi:10.1002/cam4.3351)
Supplement: Supplementary file 1 — Supplementary Material [file CAM4-9-6984-s001.docx]

**SUPLEMENTAL MATERIAL**

**QUALITY AND CAPACITY INDICATORS FOR HOSPITALIZED PEDIATRIC ONCOLOGY PATIENTS WITH CRITICAL ILLNESS: A MODIFIED DELPHI CONSENSUS**

Anita V. Arias, MD; Marcela Garza, MD; Srinivas Murthy, MD; Adolfo Cardenas, MD; Franco Diaz, MD, MBA(c); Erika Montalvo, MD; Katie R. Nielsen MD, MPH; Teresa Kortz, MD, MS; Rana Sharara-Chami, MD; Paola Friedrich, MD, MPH; Jennifer McArthur, DO; Asya Agulnik, MD, MPH

**Supplementary Appendix**

| **Section** | **Page** |
| --- | --- |
| **Table 1. Search Strategy** | **2** |
| **Table 2. Studies Included for the Development of Quality Indicator** | **3-4** |
| **Table 3. Framework of Domains and Subdomains** | **5** |
| **Table 4. Table and Map Showing Locations of the Expert Panel** | **6** |
| **Table 5. Results and Quality Indicator List During Each Consensus Round** | **7-11** |
| **Table 6. Preliminary PROACTIVE Tool** | **12-26** |

**Table 1. Search Strategy**

| DATABASE | TERMS |
| --- | --- |
| PubMed/Medline  Records Retrieved: 421 | (((((((((((((((((((((intensive care units, pediatric [MeSH Terms]) OR "pediatric intensive care units" [All Fields]) OR "pediatric critical care" [All Fields]) OR "picu" [All Fields])) AND pediatric cancer [MeSH Terms]) OR "pediatric cancer"[All Fields]) OR "pediatric oncology"[All Fields])) AND quality indicators, health care [MeSH Terms]) OR "quality indicators" [All Fields]) OR "quality markers"[All Fields]) OR "quality of care"[All Fields]) OR "quality improvement"[All Fields]) OR "capacity"[All Fields]) OR "standards of care"[All Fields]) OR "performance measures"[All Fields]) OR "performance metrics"[All Fields])) AND limited resources[MeSH Terms]) OR "limited resource countries"[All Fields]) OR "low income countries"[All Fields]) |
| Web of Science  Records Retrieved: 267 | (ALL=(intensive care units) OR ALL=(pediatric critical care) OR ALL=(PICU) AND ALL=(pediatric cancer) OR ALL=(pediatric oncology) AND ALL=(quality indicators) OR ALL=(quality of care) OR ALL=(quality improvement) OR ALL=(capacity) OR ALL=(standards of care) OR ALL=(performance metrics) AND ALL=(limited resource countries) OR ALL=(low income countries)) |
| Scopus  Records Retrieved: 61 | Pediatric intensive care units OR pediatric critical care OR PICU AND cancer AND oncology OR quality indicators OR capacity OR quality of care OR standards of care OR performance measures OR performance metrics AND limited resources |

**Table 2. Studies Included for the Development of Quality Indicator**

| AUTHORS | TITLES | COUNTRY | STUDY DESIGN | STUDY SETTING | # PICUs | # PATIENTS / PARTICIPANTS | AGE IN YEARS  (Mean$\boldsymbol{\pm}$ SD or range) | MEASURED OUTCOMES | INCLUSION CRITERIA |
| --- | --- | --- | --- | --- | --- | --- | --- | --- | --- |
| Abraham et al., 2002 | Predictors of Outcome in the Pediatric Intensive Care Units of Children with Malignancies. | Israel | Retrospective | HRS | 1 | 94 | 7.3 (2 - 21) | Intervention, severity of illness, LOS, outcomes | PICU, pediatric cancer |
| Agulnik et al., 2017 | Validation of a Pediatric Early Warning Score for Hospitalized Pediatric Oncology Patients in a Resource-Limited Setting. | Guatemala | Retrospective | RLS | 1 | 258 | 8.8 ± 5.2 | Unplanned PICU transfer, PEWS scores, mortality, LOS, severity of illness, PICU interventions | PICU, resource-limited setting, pediatric cancer, quality improvement |
| Arnim et al., 2017 | Pediatric Respiratory Support Technology and Practices: A Global Survey. | Global  (64 countries) | Survey | RLS | 270 | 295 | N/A | Respiratory support capabilities, Respiratory assessment | Pediatric critical care, resource-limited setting |
| Basnet et al., 2011 | Challenges in Setting Up Pediatric and Neonatal Intensive Care Units in a Resource-Limited Country. | Nepal | Retrospective | RLS | 1 | 20 | NR | Resources, capacity, support services, outcomes | PICU, resource-limited setting, quality improvement |
| Dalton et al., 2003 | Multicenter outcome of pediatric oncology patients requiring intensive care. | USA | Retrospective | HRS | 20 | 802 | 8.3 ± 0.2 | Resource requirements, interventions, severity of illness, LOS, outcomes | PICU, pediatric cancer |
| Dursun et al., 2009 | Prognostic Factors in Pediatric Cancer Patients Admitted to the Pediatric Intensive Care Unit. | Turkey | Retrospective | RLS | 1 | 36 | 5 (0.5 - 21) | Risk factors, neutropenia, severity of illness, interventions, LOS, outcomes. | PICU, pediatric cancer |
| Faraci et al., 2014 | Intensive care unit admission in children with malignant or nonmalignant disease: Incidence, outcome and prognostic factors: A single-center experience. | Italy | Retrospective | HRS | 1 | 54 | 8 (0 - 19) | Severity of illness, interventions, risk factors, LOS, outcomes | PICU, pediatric cancer |
| Fausser et al., 2017 | Should we pay attention to the delay before admission to a pediatric intensive care unit for children with cancer? Impact on 1-month Mortality. A report from the French Children’s Oncology Study Group, GOCE. | France | Retrospective | HRS | 6 | 224 | 7 (0 - 22) | Severity of illness, neutropenia, interventions, time of delay, LOS, outcomes | PICU, pediatric cancer |
| Garcia-Salido et al., 2015 | Respiratory Failure in Children with Hemato-Oncological Diseases admitted to the PICU: A Single-center experience. | Spain | Retro | HRS | 1 | 69 | 8 ± 5.8 | Respiratory support, severity of illness, LOS, outcomes | PICU, pediatric cancer |
| Haase et al., 2011 | Management of oncology patients admitted to the paediatric intensive care unit of a general children’s hospital – A single center analysis. | Germany | Retrospective | HRS | 1 | 66 | 10.6 ± 7.2 | Resource requirements, interventions, severity of illness, risk factors, LOS, outcomes | PICU, pediatric cancer |
| Hallahan et al., 2000 | Improved outcomes of children with malignancy admitted to a pediatric intensive care unit. | Australia | Retrospective | HRS | 1 | 150 | 5 (0 - 19) | Risk factors, severity of illness, interventions, LOS, outcomes, long term survival | PICU, pediatric cancer |
| Hernandez-Borges et al., 2017 | Set of Quality Indicators of Pediatric Intensive Care in Spain: Delphi Method Selection. | Spain | Delphi methodology | HRS | NR | 31 | N/A | 20 Quality indicators for PICU in Spain | PICU, quality indicators |
| Heying et al., 2001 | Efficacy and outcome of intensive care in pediatric oncology patients. | Germany | Retrospective | HRS | 1 | 48 | 7.8 (0 - 16.9) | Severity of illness, risk factors, interventions, LOS, outcomes | PICU, pediatric cancer |
| Keengwe et al., 1999 | Paediatric oncology and intensive care treatments: Changing trends. | UK | Retrospective | HRS | 1 | 74 | 5.4 (0.1 - 16) | Severity of illness, interventions, outcomes | PICU, pediatric cancer |
| Lee et al., 2016 | Reduction of Broad-Spectrum Antimicrobial Use in a Tertiary Children's Hospital Post Antimicrobial Stewardship Program Guideline Implementation. | USA | Retrospective | HRS | 1 | NR | NR | Guideline implementation, Antibiotic days, LOS, outcomes, costs | PICU, quality improvement |
| Murthy et al., 2015 | Intensive Care Unit Capacity in Low-Income Countries: A Systematic Review. | Global  (36 countries) | Systematic Review | RLS | 13 | NR | NR | Capacity, resources, staffing | PICU, capacity, low income countries |
| Pancera et al., 2008 | Noninvasive ventilation in immunocompromised pediatric patients: Eight years of experience in a pediatric oncology intensive care unit. | Brazil | Retrospective | RLS | 1 | 239 | 9 | Intervention, severity of illness, LOS, outcomes | PICU, pediatric cancer |
| Piastra et al., 2009 | Noninvasive pressure-support ventilation in immunocompromised children with ARDS: a feasibility study. | Italy | Prospective | HRS | 1 | 23 | 10.2 ± 4.7 | Intervention, severity of illness, risk factors, LOS, outcomes | PICU, pediatric cancer |
| Pound et al., 2008 | The Morbidity and Mortality of Pediatric Oncology Patients Presenting to the Intensive Care Unit with Septic Shock. | Canada | Retrospective | HRS | 1 | 69 | 7.8 (0 -1 7.7) | Risk factors, severity of illness, interventions, LOS, outcomes | PICU, pediatric cancer |
| Rosenthal et al., 2012 | Effectiveness of a multidimensional approach to reduce ventilator-associated pneumonia in pediatric intensive care units of 5 developing countries: International Nosocomial Infection Control Consortium findings. | 5 (Colombia, El Salvador, India, the Philippines, Turkey) | Prospective | RLS | 8 | 4339 | 1.8 ± 15.1 | Infection control program implementation, MV-days, VAP rate, LOS | PICU, resource limited settings, quality improvement |
| Scanlon et al., 2007 | Determining pediatric intensive care unit quality indicators for measuring pediatric intensive care unit safety. | USA | Survey | HRS | NR | >290 | NR | 7 quality and safety measures for PICU | PICU, quality improvement |
| Schiller et al., 2009 | Bi-Level Positive Airway Pressure Ventilation in Pediatric Oncology Patients with Acute Respiratory Failure. | Israel | Retrospective | HRS | 1 | 14 | 13.3 ± 5.3 | Respiratory failure events, interventions, images, outcomes, complications | PICU, pediatric cancer |
| Tamburro et al., 2004 | Use of the nursing acuity score in children admitted to a pediatric oncology intensive care. | USA | Prospective | HRS | 1 | 219 | 10.2 ± 0.5 | Nursing acuity scores, severity of illness, outcomes | PICU, pediatric cancer |
| Tripathi et al., 2015 | A survey on the resources and practices in pediatric critical care of resource-rich and resource-limited countries. | 34 countries (19 resource-limited) | Survey | RLS and HRS | 73 | 326 | NR (0 - 18) | Capacity, resources, staffing | PICU, resource limited settings, capacity |

*Abbreviations*: HRS (High-Resource Setting); LOS (Length of Stay); MV (Mechanical Ventilation); N/A (Not Applicable); NR (Not Reported); PEWS (Pediatric Early Warning Score); PICU (Pediatric Intensive Care Unit); RLS (Resource-Limited Setting); VAP (Ventilator Associated Pneumonia)

**Table 3. Framework of Domains and Subdomains**

| **Domains** | **Sub-domains** |
| --- | --- |
| **National Context** | National Profile & Training Programs |
| **Facility and Local Context** | Configuration of Services and Facility Utilities |
| **Finances and Resources** | Finances and Resources |
| **Personnel** | Core Team |
|  | Core Consultants |
|  | Staffing |
|  | Education, Research and Quality Improvement |
| **Service Capacity** | Service Organization |
|  | Acute Management of Critically ill PHO patients |
|  | Pediatric Outreach Team |
|  | Data Management |
|  | Standard Precautions and Infection Prevention |
|  | Guidelines and Protocols |
| **Service Integration** | Communication and Multidisciplinary Care |
|  | Parent Involvement |
|  | Quality Indicators |
| **Supportive Services** | Patients and Families Hospital Experience |
|  | General Supportive Services |
|  | General Laboratory |
|  | Imaging Services |
|  | Blood Bank/Transfusion Services |
|  | OR/Anesthesia |
| **Medication and Equipment** | Medications |
|  | Equipment and Supplies |
| **Outcomes** | Diagnosis &Outcomes |

**Table 4. Table and Map Showing Locations of the Expert Panel.** Map shows the location of participating experts during consensus round one. The table shows the number and percentage by location of the participating experts during the 3 consensus rounds.

**Table 5. Results and Quality Indicator List During Each Consensus Round.** Only indicators with high median importance (score of 7-9) in both relevance (captures key aspects in the clinical process) and actionability (can be acted upon to improve patient care) and ≥ 80% evaluator agreement were selected as part of the final set of quality indicators.

| **D** | **SD** | **QUALITY INDICATOR** | **R1** | | **R2** | | **R3** | | **Focus Group** | **Final Accepted** |
| --- | --- | --- | --- | --- | --- | --- | --- | --- | --- | --- |
|  |  |  | **Rel. (%A)** | **Act. (%A)** | **Rel. (%A)** | **Act. (%A)** | **Rel. (%A)** | **Act. (%A)** |  |  |
| **A** | **1** | Number of PICU providing care to CIPHOP | 8 (69.4%) | 7.5 (66.7%) | 8 (75.0%) | 7 (56.3%) | 8 (81.5%) | 8 (66.7%) |  |  |
|  |  | Number of trained PCCP | 8 (83.3%) | 8 (86.1%) |  |  |  |  |  | A (1) |
|  |  | A national accreditation and certification program for PICUs | 7 (69.4%) | 7 (58.3%) | 8 (75.0%) | 8 (62.5%) | 7.5 (77.8%) | 8 (66.7%) |  |  |
|  |  | National publicly funded healthcare program that provides coverage for PCI | 9 (86.1%) | 8 (77.8%) | 9 (81.3%) | 8.5 (78.1%) | 9 (92.6%) | 9 (81.5%) |  | A (2) |
|  |  | A national professional medical association for PCCM | 6 (36.1%) | 6 (36.1%) | 6 (31.3%) | 6 (37.5%) | 6 (33.3%) | 6 (33.3%) |  |  |
|  |  | Number of accredited PCCM fellowship programs | 7 (63.9%) | 7 (63.9%) | 7 (59.4%) | 7 (59.4%) | 7 (59.3%) | 7 (55.6%) |  |  |
|  |  | Accredited PCCM training/certification programs for nurses | 8 (77.8%) | 8 (72.2%) | 8 (78.1%) | 8 (87.5%) | 8 (81.5%) | 8 (85.2%) |  | A (3) |
| **B** | **2** | A designated PICU area, separated from other inpatient locations | 8.5 (83.3%) | 8 (75.0%) | 9 (87.5%) | 9 (87.5%) |  |  |  | A (4) |
|  |  | Number of PICU beds available | 7.5 (69.4%) | 7.5 (75.0%) | 7 (71.9%) | 7 (75.0%) | 8 (85.2%) | 8 (88.9%) |  | A (5) |
|  |  | Number of pediatric IMCU beds available | 7 (58.3%) | 7 (50.0%) | 7 (62.5%) | 6 (46.9%) | 7 (77.8%) | 7 (59.3%) |  |  |
|  |  | At least 1 positive/negative pressure isolation room in the PICU | 7 (52.8%) | 6 (47.2%) | 8 (65.6%) | 7 (56.3%) | 8 (70.4%) | 7 (66.7%) |  |  |
|  |  | Single-patient rooms in the PICU | 7 (63.9%) | 7 (63.9%) | 7 (65.6%) | 7 (62.5%) | 7 (77.8%) | 7 (70.4%) |  |  |
|  |  | Electricity 24/7 | 9 (80.6%) | 9 (72.2%) | 9 (90.6%) | 9 (84.4%) |  |  |  | A (6) |
|  |  | Back-up generators 24/7, in the event of power interruption | 9 (80.6%) | 8 (75.0%) | 9 (81.3%) | 9 (84.4%) |  |  |  | A (7) |
|  |  | Centrally supplied O_2_/compressed air administration systems 24/7 | 9 (83.3%) | 8 (69.4%) | 9 (90.6%) | 9 (84.4%) |  |  |  | A (8) |
|  |  | Potable water 24/7 | 9 (86.1%) | 9 (75.0%) | 9 (93.8%) | 9 (93.8%) |  |  |  | A (9) |
|  |  | Environmental temperature control system 24/7 | 7 (66.7%) | 7.5 (61.1%) | 8.5 (71.9%) | 9 (68.8%) | 9 (85.2%) | 9 (85.2%) |  | A (10) |
|  |  | Sleeping quarters for physicians within/adjacent to the PICU | 7 (66.7%) | 7.5 (69.4%) | 7 (68.8%) | 8 (75.0%) | 8 (77.8%) | 8 (77.8%) |  |  |
| **C** | **3** | % medical expenses of CIPHOP financed by the patient’s family | 7 (55.6%) | 7 (58.3%) | 7 (53.1%) | 7 (59.4%) | 7 (59.3%) | 7 (63.0%) |  |  |
|  |  | Frequency Tx limitation/modification for CIPHOP due to family’s inability to afford costs | 7.5 (55.6%) | 7 (52.8%) | 8 (71.9%) | 8 (68.8%) | 8 (74.1%) | 8 (81.5%) |  |  |
|  |  | Access to free supportive services (imaging, laboratory, blood products) for CIPHOP | 8 (61.1%) | 8 (58.3%) | 8 (62.5%) | 8 (65.6%) | 9 (77.8%) | 8 (70.4%) |  |  |
| **D** | **4** | PCCP as part of the primary team responsible for CIPHOP | 9 (83.3%) | 9 (80.6%) |  |  |  |  |  | A (11) |
|  |  | PHO physician as part of the primary team responsible for CIPHOP | 9 (77.8%) | 9 (75.0%) | 9 (81.3%) | 9 (81.3%) |  |  |  | A (12) |
|  |  | Experienced pediatrician (if no PCCP) as part of the primary team responsible for CIPHOP | 8 (75.0%) | 9 (75.0%) | 9 (81.3%) | 9 (81.3%) |  |  |  | A (13) |
|  |  | Pediatric trainees as part of the primary team responsible for CIPHOP | 7 (55.6%) | 7 (58.3%) | 7 (56.3%) | 8 (62.5%) | 7 (66.7%) | 8 (66.7%) |  |  |
|  |  | 25. PA/NP/Clinical associates as part of the primary team responsible for CIPHOP | 6 (33.3%) | 6.5 (47.2%) |  |  |  |  |  |  |
|  |  | Trained nursing staff in PCCM as part of the primary team responsible for CIPHOP | 9 (88.9%) | 9 (88.9%) |  |  |  |  |  | A (14) |
|  |  | RT as part of the primary team responsible for CIPHOP | 7 (55.6%) | 7 (52.8%) | 7.5 (59.4%) | 8 (59.4%) | 8 (74.0%) | 8 (70.4%) |  |  |
|  | **5** | PHO physician for consultation on CIPHOP | 9 (86.1%) | 8 (77.8%) | 9 (84.4%) | 9 (87.5%) |  |  |  | A (15) |
|  |  | Pediatric infectious disease physician for consultation on CIPHOP | 7 (63.9%) | 7 (61.1%) | 8 (81.3%) | 8 (71.9%) | 8 (85.2%) | 8 (74.1%) |  |  |
|  |  | Pediatric cardiologist for consultation on CIPHOP | 6 (47.2%) | 6 (47.2%) | 6 (46.9%) | 6 (46.9%) | 7 (55.6%) | 7 (59.3%) |  |  |
|  |  | Pediatric endocrinologist for consultation on CIPHOP | 6 (30.6%) | 6 (30.6%) | 6 (18.8%) | 6 (28.1%) | 6 (22.2%) | 6 (22.2%) |  |  |
|  |  | Pediatric nephrologist for consultation on CIPHOP | 7 (52.8%) | 6 (47.2%) | 6 (43.8%) | 6 (43.8%) | 6 (44.4%) | 6 (40.7%) |  |  |
|  |  | Pediatric pulmonologist for consultation on CIPHOP | 6 (41.7%) | 6 (38.9%) | 6 (21.9%) | 6 (31.3%) | 6 (22.2%) | 6 (22.2%) |  |  |
|  |  | Pediatric gastroenterologist for consultation on CIPHOP | 5 (22.2%) | 5 (27.8%) | 4 (12.5%) | 5 (21.9%) | 4 (11.1%) | 5 (14.8%) |  |  |
|  |  | Pediatric radiologist for consultation on CIPHOP | 7 (61.1%) | 7 (58.3%) | 7 (50.0%) | 7 (50.0%) | 6 (40.7%) | 6 (40.7%) |  |  |
|  |  | Pediatric interventional radiologist for consultation on CIPHOP | 6 (36.1%) | 6 (36.1%) | 6 (25.0%) | 6 (21.9%) | 6 (18.5%) | 6 (22.2%) |  |  |
|  |  | Pediatric surgeon for consultations on CIPHOP | 9 (83.3%) | 9 (83.3%) |  |  |  |  |  | A (16) |
|  |  | Pediatric neurosurgeon for consultation on CIPHOP | 8.5 (80.6%) | 8 (77.8%) | 9 (84.4%) | 9 (78.1%) | 9 (96.3%) | 9 (88.9%) |  | A (17) |
|  |  | Pediatric anesthesiologist for consultation on CIPHOP | 8 (69.4%) | 7.5 (69.4%) | 8 (65.6%) | 8 (56.3%) | 9 (74.1%) | 9 (77.8%) |  |  |
|  |  | Pediatric psychologist and/or psychiatrist for consultation on CIPHOP | 6 (47.2%) | 6 (47.2%) | 6 (37.5%) | 6 (40.6%) | 6 (29.6%) | 6 (29.6%) |  |  |
|  |  | Pediatric palliative care physician for consultation on CIPHOP | 7 (61.1%) | 7.5 (61.1%) | 7 (62.5%) | 8 (56.3%) | 8 (63.0%) | 7 (55.6%) |  |  |
|  |  | A clinical pharmacist for consultation on CIPHOP | 7 (63.9%) | 7 (66.7%) | 7 (53.1%) | 7 (68.8%) | 7 (59.3%) | 7 (74.1%) |  |  |
|  |  | A dietitian/nutritionist for consultation on CIPHOP | 6 (41.7%) | 6 (38.9%) | 6 (40.6%) | 6 (34.4%) | 6 (37.0%) | 6 (33.3%) |  |  |
|  |  | Pediatric PT/OT/rehabilitation providers for consultation on CIPHOP | 6 (41.7%) | 6 (33.3%) | 6 (43.8%) | 6 (37.5%) | 6 (33.3%) | 6 (25.9%) |  |  |
|  |  | Medical interpreter for CIPHOP who speak a different language/are hearing impaired | 7 (52.8%) | 7 (50.0%) | 6 (43.8%) | 6.5 (46.9%) | 6.5 (48.2%) | 6.5 (48.2%) |  |  |
|  | **6** | In-house PCCP staffing the PICU during the day (7d/wk) | 9 (94.4%) | 9 (88.9%) |  |  |  |  |  | A (18) |
|  |  | In-house PCCP staffing the PICU at night (7d/wk) | 8 (69.4%) | 7 (63.9%) | 8 (78.1%) | 8 (78.1%) | 9 (92.6%) | 9 (88.9%) |  | A (19) |
|  |  | In-house pediatric trainees/PA/NP staffing the PICU during the day (7d/wk) | 7 (66.7%) | 7 (66.7%) | 7 (68.8%) | 7 (68.8%) | 7 (77.8%) | 7 (81.5%) |  |  |
|  |  | In-house pediatric trainees/PA/NP staffing the PICU at night (7d/wk) | 7 (69.4%) | 7.5 (69.4%) | 7 (71.9%) | 8 (71.9%) | 7 (77.8%) | 7 (77.8%) |  |  |
|  |  | Nurses-to-patient ratio of 1:1 to 1:2 in the PICU during the day (7d/wk) | 9 (86.1%) | 9 (86.1%) |  |  |  |  |  | A (20) |
|  |  | Nurses-to-patient ratio of 1:1 to 1:2 in the PICU at night (7d/wk) | 9 (88.9%) | 9 (86.1%) |  |  |  |  |  | A (21) |
|  |  | A RT assigned solely to the PICU, during each shift (7d/wk) | 7.5 (63.9%) | 8 (69.4%) | 8 (65.6%) | 8 (65.6%) | 8 (66.7%) | 8 (66.7%) |  |  |
|  |  | A charge nurse in the PICU, during each shift (7d/wk) | 8 (75.0%) | 7 (72.2%) | 9 (84.4%) | 9 (87.5%) |  |  |  | A (22) |
|  | **7** | Formal orientation program for newly hired nurses in the PICU | 9 (83.3%) | 9 (86.1%) |  |  |  |  |  | A (23) |
|  |  | In-house educator/workshops for professional development/education of PCCN | 7.5 (66.7%) | 7 (63.9%) | 8 (71.9%) | 8 (75.0%) | 8 (81.5%) | 9 (81.5%) |  | A (24) |
|  |  | % HCP caring for CIPHOP with up-to-date pCPR certification | 9 (77.8%) | 8 (75.0%) | 9 (84.4%) | 9 (84.4%) |  |  |  | A (25) |
|  |  | Direct supervision by an attending physician of medical trainees/PA/NP in the PICU | 8 (72.2%) | 8 (72.2%) | 9 (93.8%) | 9 (90.6%) |  |  |  | A (26) |
|  |  | Free access to latest medical literature for HCP | 8 (75.0%) | 8 (80.6%) | 8 (81.3%) | 8 (90.6%) |  |  |  | A (27) |
|  |  | PICU team participation in research/QI projects to improve care for CIPHOP | 8 (77.8%) | 8 (77.8%) | 8 (84.4%) | 8 (84.4%) |  |  |  | A (28) |
| **E** | **8** | A “closed” PICU system | 7 (69.4%) | 7 (63.9%) | 8.5 (84.4%) | 8.5 (84.4%) |  |  |  | A (29) |
|  |  | An attending physician responsible for medical decisions during all shifts | 7.5 (77.8%) | 7.5 (77.8%) | 9 (87.5%) | 8 (87.5%) |  |  |  | A (30) |
|  |  | Trained HCP in performing procedures (e.g. Arterial line, CVL) | 8.5 (88.9%) | 8.5 (83.3%) |  |  |  |  |  | A (31) |
|  | **9** | A system to manage CIPHOP in the ED if no beds are available in the PICU | 7 (66.7%) | 7 (61.1%) | 8 (81.3%) | 8 (75.0%) | 8 (96.3%) | 8 (92.6%) |  | A (32) |
|  |  | Timely PICU transfer (within 4hr) of hosp. PHOP who require escalation of care | 9 (88.9%) | 9 (86.1%) |  |  |  |  |  | A (33) |
|  |  | Freq. floor management for hosp. CIPHOP due to lack of ICU beds | 8 (69.4%) | 7.5 (61.1%) | 8 (78.1%) | 8 (78.1%) | 9 (96.3%) | 9 (92.6%) | Combined | A (34) |
|  |  | Freq. vasopressor/inotrope use on the floor for PHOP due to lack of ICU beds | 7.5 (63.9%) | 7 (63.9%) | 8 (75%) | 8 (75%) | 8 (88.9%) | 8 (88.9%) |  |  |
|  |  | Freq. invasive mechanical ventilation on the floor for PHOP due to lack of ICU beds | 8 (63.9%) | 8 (61.1%) | 8 (68.8%) | 8 (59.4%) | 8 (81.5%) | 8 (77.8%) | Rescued |  |
|  | **10** | System to aid in the observation/monitoring/escalation of care for hosp. PHOP | 8 (86.1%) | 8 (83.3%) |  |  |  |  |  | A (35) |
|  |  | Availability (30 min) to a PCCP/trained HCP for consultation on hosp. PHOP | 8 (88.9%) | 8 (86.1%) |  |  |  |  |  | A (36) |
|  |  | Availability (10-15 min) to a pediatric rapid response team for hosp. PHOP | 9 (83.3%) | 9 (77.8%) | 9 (87.5%) | 9 (90.6%) |  |  |  | A (37) |
|  |  | Availability (5 min) to a pediatric code blue resuscitation team for hosp. PHOP | 9 (83.3%) | 9 (80.6%) |  |  |  |  |  | A (38) |
|  |  | Pediatric size equipment needed for resuscitation in a centralized location (“crash cart”) | 9 (86.1%) | 9 (80.6 |  |  |  |  |  | A (39) |
|  |  | A system to perform regular “crash cart” maintenance to confirm equipment and drugs | 9 (88.9%) | 9 (88.9%) |  |  |  |  |  | A (40) |
|  | **11** | Presence of an EMR system interfaced with lab results and x-ray reports | 6 (41.7%) | 6 (47.2%) | 7 (53.1%) | 7 (53.1%) | 7 (59.3%) | 7 (59.3%) |  |  |
|  | **12** | PPE as needed for management of CIPHOP (gloves, gowns, masks) | 8 (80.6%) | 7 (75.0%) | 9 (84.4%) | 9 (90.6%) |  |  |  | A (41) |
|  |  | Sterile personal attire to perform procedures as needed for management of CIPHOP | 9 (83.3%) | 8.5 (83.3%) |  |  |  |  |  | A (42) |
|  |  | Hand-washing sinks with soap and paper towels near the entrance to each CIPHOP’s room | 9 (88.9%) | 9 (88.9%) |  |  |  |  |  | A (43) |
|  |  | Access to alcohol-based hand sanitizer dispensers by the bed of each CIPHOP | 8 (80.6%) | 8.5 (83.3%) |  |  |  |  |  | A (44) |
|  | **13** | Timely assessment (5 min) of newly admitted CIPHOP to the PICU by a physician/HCP | 7 (50.0%) | 7 (58.3%) | 9 (81.3%) | 8 (81.3%) |  |  |  | A (45) |
|  |  | A written policy for PICU admission and discharge criteria for CIPHOP | 7 (52.8%) | 7 (58.3%) | 7 (65.6%) | 8 (71.9%) | 7 (77.8%) | 7 (81.5%) |  |  |
|  |  | Standardized process to confirm identity of CIPHOP prior to admin. meds/procedures | 8 (77.8%) | 8 (72.2%) | 9 (81.3%) | 9 (84.4%) |  |  |  | A (46) |
|  |  | Clinical guideline (w/latest evidence) for Mgmt./Tx of febrile neutropenia | 8 (83.3%) | 8 (77.8%) | 9 (87.5%) | 9 (87.5%) |  |  |  | A (47) |
|  |  | Clinical guideline (w/latest evidence) for Mgmt./Tx sepsis/septic shock in CIPHOP | 8 (75.0%) | 8 (75.0%) | 9 (90.6%) | 9 (90.6%) |  |  |  | A (48) |
|  |  | Clinical guideline (w/latest evidence) for Mgmt./Tx of tumor lysis syndrome | 8 (77.8%) | 8 (77.8%) | 9 (87.5%) | 9 (90.6%) |  |  |  | A (49) |
|  |  | Clinical guideline (w/latest evidence) for Mgmt./Tx of chemo related toxicities | 8 (80.6%) | 8 (72.2%) | 9 (84.4%) | 9 (84.4%) |  |  |  | A (50) |
|  |  | Clinical guideline (w/latest evidence) for Mgmt./Tx of seizure/status in CIPHOP | 8 (72.2%) | 8 (72.2%) | 8 (84.4%) | 8 (87.5%) |  |  |  | A (51) |
|  |  | Clinical guideline (w/latest evidence) for Mgmt./Tx of increased ICP in CIPHOP | 8 (72.2%) | 8 (63.9%) | 8 (84.4%) | 9 (90.6%) |  |  |  | A (52) |
|  |  | Clinical guideline (w/latest evidence) for Mgmt./Tx. of respiratory failure in CIPHOP | 8 (80.6%) | 8 (77.8%) | 9 (87.5%) | 9 (84.4%) |  |  |  | A (53) |
|  |  | A standardized process for the transport of CIPHOP | 7 (69.4%) | 8 (72.2%) | 8 (75.0%) | 8 (84.4%) | 9 (85.2%) | 9 (88.9%) |  | A (54) |
|  |  | System that allows families to request a medical order for DNR | Added by Experts | | 8 (81.3%) | 8 (71.9%) | 8 (88.9%) | 9 (92.6%) |  | A (55) |
|  |  | Process to monitor adherence to hospital policies/clinical guidelines for CIPP | Added by Experts | | 7.5 (75.0%) | 7 (71.9%) | 7 (85.2%) | 7 (81.5%) |  | A (56) |
|  |  | Standardized updated evidence-based guideline/protocols/checklists to guide care of CIPHOP | Added by Experts | | 9 (87.5%) | 9 (78.1%) | 9 (100.0%) | 9 (96.3%) |  | A (57) |
| **F** | **14** | MDT discussion of prognosis/plans/goals of care prior to admitting a CIPHOP to PICU | 7 (77.8%) | 8 (72.2%) | 8 (87.5%) | 8 (84.4%) |  |  |  | A (58) |
|  |  | MDT discussion of difficult CIPHOP cases to guide clinical/bioethical decisions | 8 (86.1%) | 8.5 (83.3%) |  |  |  |  |  | A (59) |
|  |  | Documented daily comm. between PICU/HO team regarding Mgmt./Tx of CIPHOP | 8 (83.3%) | 9 (86.1%) |  |  |  |  |  | A (60) |
|  |  | Documented daily comm. between PICU/surgical team regarding Mgmt./Tx of CIPHO | 7 (77.8%) | 8 (83.3%) | 7 (87.5%) | 8 (90.6%) |  |  |  | A (61) |
|  |  | Daily MDT patient care rounds lead by a PCCP for hospitalized CIPHOP | 7.5 (75.0%) | 8 (75.0%) | 8 (84.4%) | 9 (87.5%) |  |  |  | A (62) |
|  |  | Access to test results/reports/images to be reviewed during rounds for hosp. CIPHOP | 8.5 (83.3%) | 8 (80.6%) |  |  |  |  |  | A (63) |
|  |  | Standardized system for signing out CIPHOP between shifts among physicians | 8 (77.8%) | 8 (80.6%) | 8 (84.4%) | 8 (87.5%) |  |  |  | A (64) |
|  | **15** | Daily FCR for CIPHOP and inclusion of parents/caretakers in the decision-making process | 7 (55.6%) | 7 (55.6%) | 7.5 (59.4%) | 7.5 (53.1%) | 8 (63.0%) | 8 (66.7%) |  |  |
|  |  | Unrestricted parent visitation at all time except if not in the best interest of the child | 7 (55.6%) | 7 (58.3%) | 7 (62.5%) | 7 (65.6%) | 9 (77.8%) | 9 (81.5%) | Rescued | A (65) |
|  |  | Access to designated areas to conduct private/confidential meetings with parents of CIPHOP | 6 (47.2%) | 6.5 (50.0%) | 6 (46.9%) | 6 (46.9%) | 7 (51.9%) | 8 (59.3%) |  |  |
|  |  | Regular MDT discussion with parents regarding clinical course/futility Tx/limitation of care | 7 (69.4%) | 7 (66.7%) | 8 (75.0%) | 8 (78.1%) | 9 (92.6%) | 9 (96.3%) |  | A (66) |
|  | **16** | Surveillance system to track HAI in CIPP (including CIPHOP) | 7 (75.0%) | 9 (75.0%) | 9 (84.4%) | 9 (90.6%) |  |  |  | A (67) |
|  |  | System to monitor/audit PU/skin integrity in CIPP (including CIPHOP) | 7 (63.9%) | 7 (63.9%) | 8 (68.8%) | 8 (81.3%) | 8 (88.9%) | 8 (92.6%) |  | A (68) |
|  |  | System to monitor/audit rate unscheduled extubations in CIPP (including CIPHOP) | 7 (66.7%) | 7 (63.9%) | 8 (65.6%) | 8 (71.9%) | 8 (77.8%) | 8 (77.8%) |  |  |
|  |  | System to monitor/report abstinence-withdrawal scores in CIPP (including CIPHOP) | 7 (55.6%) | 7 (58.3%) | 8 (68.8%) | 8 (65.6%) | 8 (81.5%) | 8 (77.8%) | Rescued | A (69) |
|  |  | System to monitor/assess pain in CIPP (including CIPHOP) | 7.5 (66.7%) | 7 (69.4%) | 8 (84.4%) | 8 (81.3%) |  |  |  | A (70) |
|  |  | A surveillance system to track MDRO in hospitalized PHO patients | Added by Experts | | 8 (81.3%) | 9 (84.4%) |  |  |  | A (71) |
| **G** | **17** | Availability of a child life (play) specialist for CIPHOP | 6 (22.2%) | 6 (25.0%) | 6 (9.4%) | 6 (12.5%) | 6 (3.7%) | 6 (11.1%) |  |  |
|  |  | Availability of a chaplain or priest for CIPHOP and their families | 6 (22.2%) | 6 (27.8%) | 6 (15.6%) | 6 (25.0%) | 6 (18.5%) | 6 (22.2%) |  |  |
|  |  | Availability of a Social Worker for CIPHOP and their families | 6 (47.2%) | 6 (41.7%) | 6 (46.9%) | 6 (43.8%) | 6 (48.2%) | 7 (55.6%) |  |  |
|  |  | Availability of a meal support plan for CIPHOP and their families during the hospital stay | 6.5 (50.0%) | 7 (52.8%) | 7 (50.0%) | 7 (62.5%) | 7 (55.6%) | 7 (55.6%) |  |  |
|  |  | Housing/beds for parents of hospitalized CIPHOP next to the child or near the hospital | 6 (38.9%) | 6 (44.4%) | 6 (28.1%) | 6 (37.5%) | 6 (29.6%) | 6 (33.3%) |  |  |
|  | **18** | A system to communicate critical test results to the PICU team in a timely manner | 7 (75.0%) | 7.5 (72.2%) | 8 (81.3%) | 8 (81.3%) |  |  |  | A (72) |
|  |  | An antibiotic stewardship program to track antibiotic resistance organisms in CIPHOP | 8 (69.4%) | 7 (66.7%) | 9 (78.1%) | 8 (81.3%) | 9 (88.9%) | 9 (88.9%) |  | A (73) |
|  |  | Frequency of equipment shortage/failure affecting management of CIPHOP | 8 (75.0%) | 8 (69.4%) | 9 (84.4%) | 9 (81.3%) |  |  |  | A (74) |
|  |  | Frequency of inadequate PCCN staffing affecting management of CIPHOP | 9 (83.3%) | 9 (77.8%) | 9 (84.4%) | 9 (84.4%) |  |  |  | A (75) |
|  |  | Frequency of medication shortages affecting management of CIPHOP | 8.5 (77.8%) | 8 (77.8%) | 9 (78.1%) | 9 (75.0%) | 9 (85.2%) | 9 (81.5%) |  | A (76) |
|  |  | Frequency of blood product shortages affecting management of CIPHOP | 8.5 (86.1%) | 9 (83.3%) |  |  |  |  |  | A (77) |
|  |  | A system to monitor/prevent/address medication shortage in the PICU | 7 (77.8%) | 7 (83.3%) | 8 (78.1%) | 8 (87.5%) | 8 (88.9%) | 8 (96.3%) |  | A (78) |
|  | **19** | Point-of-care testing for pH/blood gas analysis (within 10 min) as needed for CIPHOP | 7 (61.1%) | 7 (63.9%) | 8 (71.9%) | 8 (81.3%) | 9 (85.2%) | 8 (92.6%) |  | A (79) |
|  |  | Lab services 24/7, as needed for CIPHOP | 8.5 (83.3%) | 8.5 (80.6%) |  |  |  |  |  | A (80) |
|  |  | Access to microbiology services as needed for CIPHOP | 8 (88.9%) | 8 (77.8%) | 9 (90.6%) | 9 (90.6%) |  |  |  | A (81) |
|  |  | Ability to monitor antibiotic drug levels in CIPHOP | 7 (69.4%) | 7 (66.7%) | 7 (78.1%) | 7 (75.0%) | 7 (81.5%) | 7 (85.2%) |  | A (82) |
|  |  | Ability to monitoring antineoplastic drug levels in CIPHOP | 7 (72.2%) | 7.5 (69.4%) | 7.5 (87.5%) | 8 (87.5%) |  |  |  | A (83) |
|  |  | System for drug dosing adjustments in CIPP with kidney or liver disease | 7 (72.2%) | 7 (72.2%) | 8 (78.1%) | 8 (78.1%) | 8 (88.9%) | 8 (92.6%) |  | A (84) |
|  | **20** | Availability of Plain XR (2 views) studies 24/7 when needed for CIPHOP | 9 (77.8%) | 8 (77.8%) | 9 (90.6%) | 9 (90.6%) |  |  |  | A (85) |
|  |  | Availability of Ultrasound studies 24/7 when needed for CIPHOP | 7 (61.1%) | 7 (61.1%) | 8 (71.9%) | 8 (71.9%) | 9 (74.1%) | 8 (81.5%) | Rescued | A (86) |
|  |  | Availability of CT scans 24/7 when needed for CIPHOP | 7 (75.0%) | 7 (69.4%) | 8 (75.0%) | 8 (68.8%) | 8 (88.9%) | 8 (85.2%) |  | A (87) |
|  |  | Availability of MRI studies within 24 hours for CIPHOP | 7 (63.9%) | 7 (58.3%) | 7 (59.4%) | 7 (62.5%) | 7 (70.4%) | 7 (74.1%) |  |  |
|  |  | Availability of a radiologist to review emergent imaging studies (within 1 hr) for CIPHOP | 7 (50.0%) | 7 (58.3%) | 7 (50.0%) | 7 (62.5%) | 7 (59.3%) | 7 (63.0%) |  |  |
|  |  | Availability of a pediatric radiologist to review imaging studies (within 24 hrs) for CIPHOP | 7 (63.9%) | 7 (58.3%) | 7 (68.8%) | 7 (59.4%) | 7 (85.2%) | 7 (74.1%) |  |  |
|  |  | Access to finalized radiology report for imaging studies (within 24 hrs) for CIPHOP | 7 (58.3%) | 7 (58.3%) | 7 (62.5%) | 7 (65.6%) | 7 (74.1%) | 7 (81.5%) |  |  |
|  |  | Access to portable XR devices to perform imaging studies at bedside as needed for CIPHOP | 8 (80.6%) | 8 (77.8%) | 9 (87.5%) | 9 (87.5%) |  |  |  | A (88) |
|  |  | Access to portable US devices to perform imaging studies at bedside as needed for CIPHOP | 7 (69.4%) | 7 (66.7%) | 8 (75.0%) | 8 (84.4%) | 9 (92.6%) | 8 (92.6%) |  | A (89) |
|  |  | Access to portable Echo devices for imaging studies at bedside as needed for CIPHOP | 7 (75.0%) | 7 (72.2%) | 7 (68.8%) | 7 (75.0%) | 9 (92.6%) | 7 (92.6%) |  | A (90) |
|  |  | Access to point-of-care US device to assist w/ invasive procedures/PE findings (e.g. effusion) | 7 (75.0%) | 8 (75.0%) | 8 (87.5%) | 8 (87.5%) |  |  |  | A (91) |
|  | **21** | Blood Bank Transfusion Services 24/7, providing blood products as needed for CIPHOP | 9 (86.1%) | 9 (86.1%) |  |  |  |  |  | A (92) |
|  |  | Blood type testing/cross matching pre-transfusion 24/7 as needed for CIPHOP | 9 (83.3%) | 9 (80.6%) |  |  |  |  |  | A (93) |
|  |  | Blood product screening for infectious diseases pre-transfusion 24/7 as needed for CIPHOP | 9 (83.3%) | 9 (83.3%) |  |  |  |  |  | A (94) |
|  | **22** | Access to acute surgery services 24/7 as needed for CIPHOP | 9 (86.1%) | 9 (83.3%) |  |  |  |  |  | A (95) |
|  |  | Access to an OR within 30 min., 24/7 for emergent surgeries as needed for CIPHOP | 7.5 (80.6%) | 7.5 (75.0%) | 9 (93.8%) | 9 (84.4%) |  |  |  | A (96) |
|  |  | Access to a formal post-operative surgical report (within 24 hrs) following a surgical procedure | 6.5 (50.0%) | 6 (47.2%) | 7 (53.1%) | 6 (43.8%) | 7 (55.6%) | 6 (48.2%) |  |  |
| **H** | **23** | Consistent access to first line antibiotics for CIPHOP presenting with fever and neutropenia | 9 (86.1%) | 9 (83.3%) |  |  |  |  |  | A (97) |
|  |  | Consistent access to 2^nd^/3^rd^ line antibiotics for CIPHOP with infection due to MDRO | 8 (86.1%) | 8 (80.6%) |  |  |  |  |  | A (98) |
|  |  | Consistent access to sedatives and analgesics as needed for management of CIPHOP | 9 (83.3%) | 8.5 (80.6%) |  |  |  |  |  | A (99) |
|  |  | Consistent access to antiemetics as needed for management of CIPHOP | 7 (61.1%) | 7 (61.1%) | 7 (75.0%) | 7 (78.1%) | 7 (81.5%) | 7 (85.2%) |  | A (100) |
|  |  | Consistent access to anticonvulsants as needed for management of CIPHOP | 9 (83.3%) | 9 (83.3%) |  |  |  |  |  | A (101) |
|  |  | Consistent access to IV isotonic fluids as needed for management of CIPHOP | 9 (91.7%) | 9 (88.9%) |  |  |  |  |  | A (102) |
|  |  | Consistent access to inotropes and vasoactive drugs as needed for mgmt. of CIPHOP | 9 (86.1%) | 9 (86.1%) |  |  |  |  |  | A (103) |
|  |  | Consistent access to antiarrhythmic drugs as needed for management of CIPHOP | 8 (75.0%) | 8 (75.0%) | 9 (84.4%) | 9 (87.5%) |  |  |  | A (104) |
|  |  | Consistent access to diuretics as needed for management of CIPHOP | 8 (77.8%) | 8 (77.8%) | 9 (81.3%) | 9 (78.1%) | 8 (88.9%) | 8 (85.2%) |  | A (105) |
|  |  | Consistent access to medications to treat increased ICP as needed for mgmt. of CIPHOP | 9 (80.6%) | 8 (80.6%) |  |  |  |  |  | A (106) |
|  |  | Consistent availability of parental nutrition as needed for management of CIPHOP | 7 (66.7%) | 7 (61.1%) | 8 (81.3%) | 8 (90.6%) |  |  |  | A (107) |
|  | **24** | Consistent access to continuous monitoring equipment with alarm systems at each bedside | 9 (86.1%) | 9 (88.9%) |  |  |  |  |  | A (108) |
|  |  | Consistent access to pediatric-sized equipment needed for monitoring CIPHOP (e.g. BP cuffs) | 9 (83.3%) | 9 (80.6%) |  |  |  |  |  | A (109) |
|  |  | Consistent access to critical supplies (crash carts, refrigerators for medications, respiratory equipment) within or immediately adjacent to the PICU for rapid retrieval in emergencies | 9 (83.3%) | 9 (80.6%) |  |  |  |  |  | A (110) |
|  |  | Consistent access to pediatric-sized respiratory equipment as needed for mgmt. of CIPHOP | 9 (86.1%) | 9 (83.3%) |  |  |  |  |  | A (111) |
|  |  | Consistent access to ICP monitors as needed for management of CIPHOP | 7 (63.9%) | 7 (61.1%) | 7 (65.6%) | 7 (68.8%) | 7 (81.5%) | 7 (81.5%) |  | A (112) |
|  |  | Consistent access to RRT as needed for management of CIPHOP requiring dialysis | 7 (77.8%) | 7.5 (83.3%) | 8 (84.4%) | 8 (87.5%) |  |  |  | A (113) |
|  |  | Consistent access to pediatric-sized supplies including but not limited to PIVs, IO needles, arterial/central line catheters, NG tubes, and urinary catheters as needed for CIPHOP | 9 (80.6%) | 9 (80.6%) |  |  |  |  |  | A (114) |
|  |  | Presence of an equipment maintenance and servicing department | 7 (58.3%) | 7 (56.6%) | 8 (75.0%) | 8 (81.3%) | 8 (92.6%) | 8 (88.9%) |  | A (115) |
| **I** | **25** | # PHO/BMT patients admitted to the PICU/IMCU during the past 12 months | 7 (69.4%) | 7 (55.6%) | 7 (71.9%) | 7.5 (56.3%) | 7 (81.5%) | 8 (63.0%) |  |  |
|  |  | # PHO/BMT patients admitted to the PICU/IMCU from the ED the past 12 months | 7 (52.8%) | 6.5 (47.2%) | 7 (56.3%) | 7 (53.1%) | 7 (59.3%) | 7 (55.6%) |  |  |
|  |  | # PHO/BMT patients transferred to PICU/IMCU from inpatient floors the past 12 mo | 7 (58.3%) | 7 (55.6%) | 7 (53.1%) | 7 (56.3%) | 7 (63.0%) | 7 (70.4%) |  |  |
|  |  | # Surgical PHO/BMT patients that required post-operative PICU care the past 12 mo | 7 (58.3%) | 6 (47.2%) | 7 (62.5%) | 7 (56.3%) | 7 (74.1%) | 7 (63.0%) |  |  |
|  |  | # PHO/BMT patients admitted to PICU/IMCU for sepsis during the past 12 months | 7 (69.4%) | 7 (58.3%) | 7.5 (75.0%) | 7 (65.6%) | 7 (81.5%) | 7 (70.4%) |  |  |
|  |  | # PHO/BMT patients admitted to PICU/IMCU for respiratory failure the past 12 mo | 7 (69.4%) | 7 (55.6%) | 7 (71.9%) | 7 (65.6%) | 7 (85.2%) | 7 (77.8%) |  |  |
|  |  | # PHO/BMT patients admitted to PICU/IMCU for MODS during the past 12 months | 7 (63.9%) | 7 (52.8%) | 7 (71.9%) | 7 (62.5%) | 7 (74.1%) | 7.5 (70.4%) |  |  |
|  |  | Median LOS in PICU/IMCU for PHO/BMT patients during the past 12 months | 7 (61.1%) | 6 (47.2%) | 7 (59.4%) | 7 (56.3%) | 7 (66.7%) | 7 (74.1%) |  |  |
|  |  | # PHO/BMT patients requiring mechanical ventilator support during the past 12 mo | 7 (61.1%) | 6 (47.2%) | 7 (71.9%) | 7 (59.4%) | 7 (81.5%) | 7.5 (63.0%) |  |  |
|  |  | A system to aid in the prediction of illness severity and mortality in the PICU (e.g. PRISM) | 7 (52.8%) | 7 (63.9%) | 7 (65.6%) | 7.5 (71.9%) | 8 (70.4%) | 8 (77.8%) |  |  |
|  |  | Collaboration with other regional/international centers to compare/benchmark outcomes | 7 (55.6%) | 8 (63.9%) | 7 (59.4%) | 8 (71.9%) | 8 (74.1%) | 9 (85.2%) | Rescued | A (116) |
|  |  | System to report/analyze major patient AD occurring in CIPP/PHO patients | 8 (66.7%) | 9 (75.0%) | 9 (71.9%) | 9 (87.5%) | 9 (88.9%) | 9 (96.3%) |  | A (117) |
|  |  | Regular MDT meetings to review morbidity/mortality cases, unexpected transfers, clinical incidents and near misses for CIPP/PHO patients | 8 (66.7%) | 8 (72.2%) | 9 (81.3%) | 9 (81.3%) |  |  |  | A (118) |
|  |  | Mortality rate of PHO/BMT patient in the PICU/IMCU during the past 12 months. | 9 (63.9%) | 7 (61.1%) | 9 (68.8%) | 9 (65.6%) | 9 (77.8%) | 9 (85.2%) | Rescued/ Combined | A (119) |
|  |  | A patient data registry that includes mortality of hosp. PHO/BMT patients in the PICU/IMCU | 7 (63.9%) | 7.5 (72.2%) | 7 (62.5%) | 8 (78.1%) | 8 (66.7%) | 8 (88.9%) |  |  |

| **Scoring** | |
| --- | --- |
| **Accepted** | Median score of 7-9 and ≥ 80% evaluator agreement |
| **Uncertain** | Median score 4-9 and < 80% evaluator agreement |
| **Unimportant** | Median score (1-3) |
| **Other** | Rescued and combined quality indicators |

**Abbreviations**: Act. (Actionability); AE (Adverse Events); BMT (Bone Marrow Transplant); CIPHOP (Critically Ill Pediatric Hematology-Oncology Patients); CIPP (Critically Ill Pediatric Patients); CVL (Central Venous line); D (Domain); DNR (Do Not Resuscitate); ED (Emergency Department); EMR (Electronic Medical Record); FCR (Family Center Rounds); HAI (Hospital Acquired Infections); HCP (Healthcare Providers); ICP (Intracranial Pressure); IMCU (Intermediate Medical Care Unit); LOS (Length of Stay); MDRO (Multi-Drug Resistant Organisms); MDT (Multidisciplinary Team); NP (Nurse Practitioners); OT (Occupational Therapists); PA (Physician Assistants); PCCM (Pediatric Critical Care Medicine); PCCN (Pediatric Critical Care Nurses); PCCP (Pediatric Critical Care Physicians); PCI (Pediatric Critical Illness); pCPR (Pediatric Cardiopulmonary Resuscitation); PE (Physical Exam); PHO (Pediatric Hematology-Oncology); PHOP (Pediatric Hematology-Oncology Patients); PICU (Pediatric Intensive Care Unit); PPE (Personal Protective Equipment); PT (Physical Therapists); PU (Pressure Ulcers); QI (Quality Improvement); R (Round); Rel. (Relevance); RRT (Renal Replacement Therapy); RT (Respiratory Therapists); SD (Subdomain); Tx (Treatment); %A (Percentage of Evaluator Agreement); 24/7 (24 hours a day, 7 days a week); 7d/wk (7 days a week).

**Table 6. Preliminary PROACTIVE Tool.** Preliminary version of the PROACTIVE tool, it shows the selected indicators in questions format with their answer choices.

| **Domain** | **Sub-domain** | **Questions** | **Answer Choices** | |
| --- | --- | --- | --- | --- |
| **National Context** | National Profile & Training Programs | How many trained Pediatric Critical Care Physicians are currently practicing in your country? (Please provide a best estimate and mention on data sources, if available in the comments) | *__________* | |
|  |  |  | *Do not know/no information available* | |
|  |  |  | *Comments:* | |
|  |  | Does your country have a national publicly funded healthcare program (endorsed by the Ministry of Health) that provides coverage for pediatric critical illness? | *No funding mechanism exists AND no significant input from non-public sources; families heavily burden by out of pocket expenses* | |
|  |  |  | *No funding mechanism exists, but non-public sources are available; families are highly dependent on these alternate sources of funding for coverage of direct and indirect costs* | |
|  |  |  | *Funding mechanism for most direct costs exists, but families remain highly dependent on alternate sources for coverage of indirect costs* | |
|  |  |  | *Most direct costs of care are covered; residual indirect costs exist and can cause material hardship, but pose low additional risk of family bankruptcy* | |
|  |  | Does your country have an accredited pediatric critical care training or certification program for nurses? | *Yes (if yes, please describe: ____________)* | |
|  |  |  | *No (if No, where do interested nurses typically obtain training? _______)* | |
|  |  |  | *Do not know* | |
| **Facility and Local Context** | Configuration of services and Facility Utilities | Is there a designated Pediatric Intensive Care Unit (designated area within a hospital), separated from other inpatient locations (e.g. general ward) at your facility? | *Yes* | |
|  |  |  | *No (if No, where are critically ill pediatric patients managed?)*  *General Pediatric ward*  *Combined pediatric/adult ward*  *Adult ICU*  *Other, explain: ______* | |
|  |  |  | *Do not know* | |
|  |  | What is the total number of pediatric intensive care beds available in your facility? | *≤ 10* | *11-20* |
|  |  |  | *21-30* | *>31* |
|  |  |  | *Do not know* | |
|  |  | Does your facility have electricity available 24 hours a day, 7 days a week? | *Almost always* | *Frequently* |
|  |  |  | *Sometimes* | *Infrequently* |
|  |  |  | *Almost Never* | *Not available* |
|  |  |  | *if NOT almost always, are the breaks in electricity ≤ 2hrs per day?*  *Yes ii. No iii. Do not know* | |
|  |  | Does your facility have back-up generators available 24 hours a day, 7 days a week to provide electricity in the event of power interruption? | *Almost always* | *Frequently* |
|  |  |  | *Sometimes* | *Infrequently* |
|  |  |  | *Almost Never* | *Not available* |
|  |  | Does your facility have centrally supplied oxygen and/or compressed air administration systems available 24 hours a day, 7 days a week? | *Almost always* | *Frequently* |
|  |  |  | *Sometimes* | *Infrequently* |
|  |  |  | *Almost Never* | *Not available* |
|  |  |  | *if NOT almost always, are the breaks in supply ≤ 2hrs per day?*  *Yes ii. No iii. Do not know* | |
|  |  | Does your facility have potable water available 24 hours a day, 7 days a week? | *Almost always* | *Frequently* |
|  |  |  | *Sometimes* | *Infrequently* |
|  |  |  | *Almost Never* | *Not available* |
|  |  |  | *if NOT almost always, are the breaks in supply ≤ 2hrs per day?*  *Yes ii. No iii. Do not know* | |
|  |  | Does your facility have an environmental temperature control system (e.g. heater and air conditioner) available 24hrs per day, 7 days a week? | *Almost always* | *Frequently* |
|  |  |  | *Sometimes* | *Infrequently* |
|  |  |  | *Almost Never* | *Not available* |
|  |  |  | *if NOT almost always, are the breaks in supply ≤ 2hrs per day?*  *Yes ii. No iii. Do not know* | |
| **Personnel**  **Personnel** | Core Team | Is a pediatric critical care physician available as part of the primary medical team responsible for the care of critically ill PHO patients? (provider dedicates time and is invested in the care of these patients) | *Almost Always* | *Frequently* |
|  |  |  | *Sometimes/Moderate availability* | *Infrequently/Limited availability* |
|  |  |  | *Almost Never* | *Not available* |
|  |  |  | *Do not know* | |
|  |  | Is a pediatric hematology/oncology physician available as part of the primary medical team responsible for the care of critically ill PHO patients? (provider dedicates time and is invested in the care of these patients) | *Almost Always* | *Frequently* |
|  |  |  | *Sometimes/Moderate availability* | *Infrequently/Limited availability* |
|  |  |  | *Almost Never* | *Not available* |
|  |  |  | *Do not know* | |
|  |  | Is a pediatrician with experience managing critical ill patients (if no pediatric intensivist) available as part of the primary medical team responsible for the care of critically ill PHO patients? (provider dedicates time and is invested in the care of these patients) | *Almost Always* | *Frequently* |
|  |  |  | *Sometimes/Moderate availability* | *Infrequently/Limited availability* |
|  |  |  | *Almost Never* | *Not available* |
|  |  |  | *Do not know* | |
|  |  | Is nursing staff formally trained in pediatric critical care available as part of the primary medical team responsible for the care of critically ill PHO patients? (provider dedicates time and is invested in the care of these patients) | *Almost Always* | *Frequently* |
|  |  |  | *Sometimes/Moderate availability* | *Infrequently/Limited availability* |
|  |  |  | *Almost Never* | *Not available* |
|  |  |  | *Do not know* | |
|  | Core Consultants | How often are pediatric hematology/oncology physician available for consults on critically ill PHO patients? (provider dedicates time and is invested in the care of these patients) | *Almost Always* | *Frequently* |
|  |  |  | *Sometimes/Moderate availability* | *Infrequently/Limited availability* |
|  |  |  | *Almost Never* | *Not available* |
|  |  |  | *Do not know* | |
|  |  | How often are pediatric surgeons available for consults on critically ill PHO patients? (provider dedicates time and is invested in the care of these patients) | *Almost Always* | *Frequently* |
|  |  |  | *Sometimes/Moderate availability* | *Infrequently/Limited availability* |
|  |  |  | *Almost Never* | *Not available* |
|  |  |  | *Do not know* | |
|  |  | How often are pediatric neurosurgeons available for consults on critically ill PHO patients? (provider dedicates time and is invested in the care of these patients) | *Almost Always* | *Frequently* |
|  |  |  | *Sometimes/Moderate availability* | *Infrequently/Limited availability* |
|  |  |  | *Almost Never* | *Not available* |
|  |  |  | *Do not know* | |
|  | Staffing | How often are pediatric critical care physicians in house (in the hospital) staffing the PICU (or area where critically ill PHO patients are treated) during day shifts, 7 days a week? | *Almost Always* | *Frequently* |
|  |  |  | *Sometimes/Moderate availability* | *Infrequently/Limited availability* |
|  |  |  | *Almost Never* | *Not available* |
|  |  |  | *Do not know* | |
|  |  | How often are pediatric critical care physicians in-house (in the hospital) staffing the PICU (or area where critically ill PHO patients are treated) during night shifts, 7 days a week? | *Almost Always* | *Frequently* |
|  |  |  | *Sometimes/Moderate availability* | *Infrequently/Limited availability* |
|  |  |  | *Almost Never* | *Not available* |
|  |  |  | *Do not know* | |
|  |  | What is the Nurse-to-patient ratio in the PICU (or area where critically ill PHO patients are treated) during day shifts, 7 days a week | *1: 1-2* | *1: 3-4* |
|  |  |  | *1: 5-6* | *1: >7, please describe: _______* |
|  |  |  | *Do not know* | |
|  |  | What is the nurse-to-patient ratio in the PICU (or area where critically ill PHO patients are treated) during night shifts, 7 days a week? | *1: 1-2* | *1: 3-4* |
|  |  |  | *1: 5-6* | *1: >7, please describe: ______* |
|  |  |  | *Do not know* | |
|  |  | How often is there a charge nurse who is responsible for the organization, management, supervision and quality of nursing practices in the PICU (or area where critically ill PHO patients are treated), available during each shift (day and night), 7 days a week? | *Almost Always* | *Frequently* |
|  |  |  | *Sometimes/Moderate availability* | *Infrequently/Limited availability* |
|  |  |  | *Almost Never* | *Not available* |
|  |  |  | *Do not know* | |
|  | Education, Research and Quality Improvement | Does your facility have a formal orientation program focused on pediatric critical care for newly hired nurses in the PICU (or area where critically ill pediatric patients are treated)? | *Yes* | |
|  |  |  | *No* | |
|  |  |  | *Do not know* | |
|  |  | How often is there an in-house (in the hospital) educator or workshops for the professional development, training and education of pediatric critical care nurses available at your facility? | *Almost Always* | *Frequently* |
|  |  |  | *Sometimes/Moderate availability* | *Infrequently/Limited availability* |
|  |  |  | *Almost Never* | *Not available* |
|  |  |  | *Do not know* | |
|  |  | What is the percentage of healthcare providers caring for critically ill PHO patients who have up-to-date pediatric cardiorespiratory resuscitation certification (e.g. PALS, BLS)? (Please provide a best estimate in this field and comment on data sources in the comments if available) | *_________ (percentage)* | |
|  |  |  | *Do not know/no information available* | |
|  |  |  | *Comments:* | |
|  |  | How often do medical trainees (fellows and residents), physician assistants, clinical associates and nurse practitioners work under the direct supervision of an attending physician when caring for critically ill PHO patient in the PICU (or area where critically ill PHO patients are treated)? | *Almost Always* | *Frequently* |
|  |  |  | *Sometimes* | *Infrequently* |
|  |  |  | *Almost Never* | *Do not know* |
|  |  | How often is there free access to resources for reviewing the latest medical literature for healthcare providers (e.g. PubMed, UpToDate) at your facility? | *Almost Always* | *Frequently* |
|  |  |  | *Sometimes/Moderate availability* | *Infrequently/Limited availability* |
|  |  |  | *Almost Never* | *Not available* |
|  |  |  | *Do not know* | |
|  |  | Does the pediatric critical care team participate in research or quality improvement projects designed to improve the care of critically ill PHO patients? | *No* | |
|  |  |  | *Yes (check all that apply)*  *Education*  *Quality improvement or patient care delivery*  *Research*  *Other (explain): ________* | |
|  |  |  | *Do not know* | |
| **Service**  **Capacity**  **Service**  **Capacity**  **Service Capacity** | Service Organization | Does your facility have a “closed” PICU system, where the primary responsibility and management of admitted critically ill PHO patients is transferred to the pediatric critical care physician? | *Yes* | |
|  |  |  | *No, please describe:* | |
|  |  |  | *Do not know* | |
|  |  | How often is an attending physician (not trainees) primarily responsible for the medical decisions involving critically ill PHO patients, during all shifts, 7 days a week? | *Almost Always* | *Frequently* |
|  |  |  | *Sometimes* | *Infrequently* |
|  |  |  | *Almost Never* | *Do not know* |
|  |  | How often is a healthcare provider (e.g. physician, fellow, nurse practitioner) trained in performing procedures, including but not limited to arterial line, central line and chest tube placement, available as needed for the care of critically ill PHO pediatric patients at your facility? | *Almost Always* | *Frequently* |
|  |  |  | *Sometimes* | *Infrequently* |
|  |  |  | *Almost Never* | *Do not know* |
|  | Acute Management of Critically ill PHO patients | Is there a system in place to manage critically ill PHO patients in the Emergency Department (ED) if no beds are available in the PICU (or area where critically ill PHO patients are treated) at your facility? | *Yes* | |
|  |  |  | *No, please describe:* | |
|  |  |  | *Do not know* | |
|  |  | How often are hospitalized PHO patients who require escalation of care transferred in a timely manner (within 4 hours) to the PICU (or area where critically ill PHO patients are treated) from other in-patient hospital units (e.g. general floor, PHO unit)? | *Almost Always* | *Frequently* |
|  |  |  | *Sometimes* | *Infrequently* |
|  |  |  | *Almost Never* | *Do not know* |
|  |  |  | *How long does it typically take to transfer patients to the PICU?*  *<1 hr. ii. 1-3 hrs. iii. 4-8 hrs. iv. >8hrs.* | |
|  |  | A. How often are hospitalized PHO patients who develop critical illness (respiratory failure, hypotension, etc.) managed on the floor/ward/inpatient care area (rather than in a PICU) due to lack of ICU beds? | *Almost Always* | *Frequently* |
|  |  |  | *Sometimes* | *Infrequently* |
|  |  |  | *Almost Never* | *Do not know* |
|  |  | B. How often are vasopressors or inotropes used on the floor/ward/inpatient care area (rather than in a PICU) for hospitalized PHO patients requiring vasoactive infusions, due to lack of ICU beds? | *Almost Always* | *Frequently* |
|  |  |  | *Sometimes* | *Infrequently* |
|  |  |  | *Almost Never* | *Do not know* |
|  |  | C. How often is invasive mechanical ventilation used on the floor/ward/inpatient care area (rather than in a PICU) for hospitalized PHO patients with respiratory failure due to lack of ICU beds? | *Almost Always* | *Frequently* |
|  |  |  | *Sometimes* | *Infrequently* |
|  |  |  | *Almost Never* | *Do not know* |
|  | Pediatric Outreach Team | Does your facility have a system in place to aid in the observation, monitoring and escalation of care (e.g. PEWS—Pediatric Early Warning System) for hospitalized PHO patients? | *Yes* | |
|  |  |  | *No* | |
|  |  |  | *Do not know* | |
|  |  | How often is a pediatric critical care physician or trained healthcare provider (fellow, nurse practitioner, physician assistant) available for consultation (within 30 minutes) on hospitalized PHO patients presenting with acute clinical changes or deterioration? | *Almost Always* | *Frequently* |
|  |  |  | *Sometimes/Moderate availability* | *Infrequently/Limited availability* |
|  |  |  | *Almost Never* | *Not available* |
|  |  |  | *Do not know* | |
|  |  | How often is a pediatric rapid response team available (within 10-15 minutes) for hospitalized PHO patients who develop signs of acute clinical deterioration? | *Almost Always* | *Frequently* |
|  |  |  | *Sometimes/Moderate availability* | *Infrequently/Limited availability* |
|  |  |  | *Almost Never* | *Not available* |
|  |  |  | *Do not know* | |
|  |  | How often is a pediatric code blue resuscitation team available (within 5 minutes) for hospitalized PHO patients with signs of cardiorespiratory arrest? | *Almost Always* | *Frequently* |
|  |  |  | *Sometimes/Moderate availability* | *Infrequently/Limited availability* |
|  |  |  | *Almost Never* | *Not available* |
|  |  |  | *Do not know* | |
|  |  | How often is pediatric size equipment (e.g. paddles, endotracheal tubes) easily accessible in a centralized location (“crash cart”) when needed for acute resuscitation of critically ill pediatric patients (including critically ill PHO patients) at your facility? | *Almost Always* | *Frequently* |
|  |  |  | *Sometimes/Moderate availability* | *Infrequently/Limited availability* |
|  |  |  | *Almost Never* | *Not available* |
|  |  |  | *Do not know* | |
|  |  | How often does your facility perform regular “crash cart” maintenance inventory to confirm accessibility to appropriate equipment and emergency drugs? | *Almost Always* | *Frequently* |
|  |  |  | *Sometimes* | *Infrequently* |
|  |  |  | *Almost Never* | *Not available* |
|  |  |  | *Do not know* | |
|  | Standard Precautions and Infection Prevention | How often is personal protective equipment accessible for the management of critically ill PHO patients? (please indicate which items are available at your facility) | *Examination Globes*  *Almost Always*  *Frequently*  *Sometimes/Moderate availability*  *Infrequently/Limited availability*  *Almost Never*  *Not available*  *Do not know* | *Gowns*  *Almost Always*  *Frequently*  *Sometimes/Moderate availability*  *Infrequently/Limited availability*  *Almost Never*  *Not available*  *Do not know* |
|  |  |  | *Masks*  *Almost Always*  *Frequently*  *Sometimes/Moderate availability*  *Infrequently/Limited availability*  *Almost Never*  *Not available*  *Do not know* | *Eye Shields*  *Almost Always*  *Frequently*  *Sometimes/Moderate availability*  *Infrequently/Limited availability*  *Almost Never*  *Not available*  *Do not know* |
|  |  | How often are *sterile* personal protective attire items regularly available to perform procedures (e.g. line placement) on critically ill PHO patients? (please indicate which items are available at your facility) | *Sterile Gloves*  *Almost Always*  *Frequently*  *Sometimes/Moderate availability*  *Infrequently/Limited availability*  *Almost Never*  *Not available*  *Do not know* | *Sterile Gowns*  *Almost Always*  *Frequently*  *Sometimes/Moderate availability*  *Infrequently/Limited availability*  *Almost Never*  *Not available*  *Do not know* |
|  |  |  | *Hats*  *Almost Always*  *Frequently*  *Sometimes/Moderate availability*  *Infrequently/Limited availability*  *Almost Never*  *Not available*  *Do not know* |  |
|  |  | How often is there adequate access to hand-washing sinks equipped with soap and paper towels near the entrance to each critically ill PHO patient’s room in the PICU (or area where critically ill PHO patients are treated)? | *Almost Always* | *Frequently* |
|  |  |  | *Sometimes/Moderate availability* | *Infrequently/Limited availability* |
|  |  |  | *Almost Never* | *Not available* |
|  |  |  | *Do not know* | |
|  |  | How often is there adequate access to alcohol-based hand sanitizer dispensers by the bed of each critically ill PHO patient in the PICU (or area where critically ill PHO patients are treated)? | *Almost Always* | *Frequently* |
|  |  |  | *Sometimes/Moderate availability* | *Infrequently/Limited availability* |
|  |  |  | *Almost Never* | *Not available* |
|  |  |  | *Do not know* | |
|  | Guidelines and Protocols | How often are newly admitted critically ill PHO patients assessed (within 5 min) upon arrival to the PICU (or area where critically ill PHO patients are treated) by a physician or trained healthcare provider (nurse practitioner, physician assistant)? | *Almost Always* | *Frequently* |
|  |  |  | *Sometimes* | *Infrequently* |
|  |  |  | *Almost Never* | *Do not know* |
|  |  | Does your facility have a standardized process to confirm the identity of critically ill PHO patients (e.g., unique patient identifiers) prior to the administration of medications (e.g. chemotherapy) and/or procedures? | *Yes* | |
|  |  |  | *No* | |
|  |  |  | *Do not know* | |
|  |  | Is there a clinical guideline (updated with the latest medical evidence) for the management and treatment of febrile neutropenia in critically ill PHO patients | *Yes* | |
|  |  |  | *No* | |
|  |  |  | *Do not know* | |
|  |  | Is there a clinical guideline (updated with the latest medical evidence) for the management and treatment of sepsis/septic shock in critically ill PHO patients? | *Yes* | |
|  |  |  | *No* | |
|  |  |  | *Do not know* | |
|  |  | Is there a clinical guideline (updated with the latest medical evidence) for the management and treatment of tumor lysis syndrome in critically ill PHO patients? | *Yes* | |
|  |  |  | *No* | |
|  |  |  | *Do not know* | |
|  |  | Is there a clinical guideline (updated with the latest medical evidence) for the management and treatment of chemotherapy related toxicities (e.g. hemorrhagic cystitis, methotrexate toxicity) in critically ill PHO patients? | *Yes* | |
|  |  |  | *No* | |
|  |  |  | *Do not know* | |
|  |  | Is there a clinical guideline (updated with the latest medical evidence) for the management and treatment of seizure and status epilepticus in critically ill pediatric patients (including critically ill PHO patients) | *Yes* | |
|  |  |  | *No* | |
|  |  |  | *Do not know* | |
|  |  | Is there a clinical guideline (updated with the latest medical evidence) for the management and treatment of increased intracranial pressure in critically ill pediatric patients (including critically ill PHO patients)? | *Yes* | |
|  |  |  | *No* | |
|  |  |  | *Do not know* | |
|  |  | Is there a clinical guideline (updated with the latest medical evidence) for the management and treatment of respiratory failure in critically ill PHO patients? | *Yes* | |
|  |  |  | *No* | |
|  |  |  | *Do not know* | |
|  |  | Is there a standardized process for the transport of critically ill PHO patients (within the hospital, to the PICU from other facilities, and from the PICU to other facilities)? | *Yes* | |
|  |  |  | *No* | |
|  |  |  | *Do not know* | |
|  |  | Is there a system that allows families to request a medical order for DNR (Do not resuscitate) allowing the medical team to withhold cardiopulmonary resuscitation of a dying pediatric patient at your facility? | *Yes* | |
|  |  |  | *No* | |
|  |  |  | *Do not know* | |
|  |  | Is there a standardized process to monitor adherence to hospital policies and clinical guidelines for critically ill pediatric patient at your facility? | *Yes* | |
|  |  |  | *No* | |
|  |  |  | *Do not know* | |
|  |  | Are there standardized updated evidence-based guidelines, protocols and checklists to guide the clinical care of critically ill PHO patients at your facility (e.g. nausea/vomiting, constipation, pain)? | *Yes* | |
|  |  |  | *No* | |
|  |  |  | *Do not know* | |
| **Service Integration** | Communication  and  Multidisciplinary  Care | How often are multidisciplinary team discussions regarding prognosis, plan of care, and goals of care, conducted *prior* to admitting a critically ill PHO patient to the PICU? | *Almost Always* | *Frequently* |
|  |  |  | *Sometimes* | *Infrequently* |
|  |  |  | *Almost Never* | *Do not know* |
|  |  | How often are multidisciplinary team discussions regarding difficult critically ill PHO patient cases conducted (when indicated) to guide clinical or bioethical decisions? | *Almost Always* | *Frequently* |
|  |  |  | *Sometimes* | *Infrequently* |
|  |  |  | *Almost Never* | *Do not know* |
|  |  | How often is there daily documented communication between the PICU and the hematology/oncology team regarding management and treatment of critically ill PHO patients? | *Almost Always* | *Frequently* |
|  |  |  | *Sometimes* | *Infrequently* |
|  |  |  | *Almost Never* | *Do not know* |
|  |  | How often is daily documented communication occurring between the PICU and the surgical team regarding surgical procedures and post operatively management of critically ill PHO patients requiring surgical care (pre/post-op)? | *Almost Always* | *Frequently* |
|  |  |  | *Sometimes* | *Infrequently* |
|  |  |  | *Almost Never* | *Do not know* |
|  |  | How often are daily multidisciplinary patient care rounds for hospitalized critically ill PHO patients lead by a pediatric critical care physician? | *Almost Always* | *Frequently* |
|  |  |  | *Sometimes* | *Infrequently* |
|  |  |  | *Almost Never* | *Do not know* |
|  |  | How often are relevant test results, reports, and imaging studies available for review during daily rounds for hospitalized critically ill PHO patients? | *Almost Always* | *Frequently* |
|  |  |  | *Sometimes* | *Infrequently* |
|  |  |  | *Almost Never* | *Do not know* |
|  |  | Is there a standardized system for signing out critically ill PHO patients between shifts, to give continuity of care every time responsibilities are transferred between physicians? | *Yes* | |
|  |  |  | *No* | |
|  |  |  | *Do not know* | |
|  | Parent Involvement | Does your facility allow unrestricted parent visitation for critically ill PHO patients at all time except when this is not in the best interest of the child and the family (e.g. during sterile procedures)? | *Yes* | |
|  |  |  | *No* | |
|  |  |  | *Do not know* | |
|  |  | How often are multidisciplinary team discussions with parents of critically ill PHO patients (care conferences) held to discuss the clinical course and, when appropriate, futility of treatment, end of life or limitation of care? (Care conferences are meetings between the family, ICU and hematology-oncology team to discuss a patient´s clinical course) | *Almost Always* | *Frequently* |
|  |  |  | *Sometimes* | *Infrequently* |
|  |  |  | *Almost Never* | *Do not know* |
|  | Quality Indicators | Does your facility have a surveillance system to track hospital-acquired infections (e.g. CLABSI, VAP, CAUTI) in critically ill pediatric patients (including critically ill PHO patients)? | *Yes* | |
|  |  |  | *No* | |
|  |  |  | *Do not know* | |
|  |  | Does your facility have a system to monitor and audit pressure ulcers (PU) and skin integrity in critically ill pediatric patients (including critically ill PHO patients)? | *Yes* | |
|  |  |  | *No* | |
|  |  |  | *Do not know* | |
|  |  | Does your facility have a standardized system to monitor and report abstinence and withdrawal scores (e.g. WAT scores) in critically ill pediatric patients (including critically ill PHO patients)? | *Yes* | |
|  |  |  | *No* | |
|  |  |  | *Do not know* | |
|  |  | Does your facility have a standardized system to monitor and assess pain (e.g. Wong-Baker FACES pain rating scale) in critically ill pediatric patients (including critically ill PHO patients)? | *Yes* | |
|  |  |  | *No* | |
|  |  |  | *Do not know* | |
|  |  | Does your facility have a surveillance system to track multi-drug resistance organisms (MDRO) in hospitalized PHO patients? | *Yes* | |
|  |  |  | *No* | |
|  |  |  | *Do not know* | |
| **Supportive Services**  **Supportive Services**  **Supportive Services** | Gen. Supportive Services | Does your facility have a system or process to communicate critical test results to the critical care team in a timely manner? | *Yes* | |
|  |  |  | *No* | |
|  |  |  | *Do not know* | |
|  |  | Does your facility have an antibiotic stewardship to track antibiotic resistance organisms in critically ill pediatric patients (including critically ill PHO patients)? | *Yes* | |
|  |  |  | *No* | |
|  |  |  | *Do not know* | |
|  |  | How often do equipment shortages and/or failures affect the management of critically ill PHO patients? | *Almost Always* | *Frequently* |
|  |  |  | *Sometimes* | *Infrequently* |
|  |  |  | *Almost Never* | *Do not know* |
|  |  | How often does inadequate pediatric critical care nurse staffing affect the management of critically ill PHO patients? | *Almost Always* | *Frequently* |
|  |  |  | *Sometimes* | *Infrequently* |
|  |  |  | *Almost Never* | *Do not know* |
|  |  | How often do medication shortages affect the management of critically ill PHO patients? | *Almost Always* | *Frequently* |
|  |  |  | *Sometimes* | *Infrequently* |
|  |  |  | *Almost Never* | *Do not know* |
|  |  | How often do blood product (packed red blood cells, platelets, fresh frozen plasma, etc.) shortages affect the management of critically ill PHO patients? | *Almost Always* | *Frequently* |
|  |  |  | *Sometimes* | *Infrequently* |
|  |  |  | *Almost Never* | *Do not know* |
|  |  | Is there a standard system to monitor, prevent, and/or address medication shortages in the PICU (or area where critically ill PHO patients are managed) at your facility? | *Yes* | |
|  |  |  | *No* | |
|  |  |  | *Do not know* | |
|  | Gen Lab | How often is point-of-care testing available for pH and blood gas analysis (within 10 minutes) as needed for critically ill PHO patients)? | *Almost Always* | *Frequently* |
|  |  |  | *Sometimes/Moderate availability* | *Infrequently/Limited availability* |
|  |  |  | *Almost Never* | *Not available* |
|  |  |  | *Do not know* | |
|  |  | How often are the following laboratory services available as needed for critically ill PHO patients at your facility? | *Blood cell count (CBC + differential)*  *Almost Always (24/7)*  *Frequently*  *Sometimes/Moderate availability*  *Infrequently/Limited availability*  *Almost Never*  *Not available*  *Do not know* | *Complete metabolic panel*  *Almost Always (24/7)*  *Frequently*  *Sometimes/Moderate availability*  *Infrequently/Limited availability*  *Almost Never*  *Not available*  *Do not know* |
|  |  |  | *Coagulation studies (INR, PTT)*  *Almost Always (24/7)*  *Frequently*  *Sometimes/Moderate availability*  *Infrequently/Limited availability*  *Almost Never*  *Not available*  *Do not know* | *LDH, Ferritin*  *Almost Always (24/7)*  *Frequently*  *Sometimes/Moderate availability*  *Infrequently/Limited availability*  *Almost Never*  *Not available*  *Do not know* |
|  |  |  | *Urine analysis*  *Almost Always (24/7)*  *Frequently*  *Sometimes/Moderate availability*  *Infrequently/Limited availability*  *Almost Never*  *Not available*  *Do not know* | *In general, what is the turn-around time (reporting time) for STAT (emergency) laboratory test results?*  *< 1 hr.*  *1-4 hrs.*  *4-8 hrs.*  *8-12 hrs.*  *>12 hrs.* |
|  |  | How often are microbiology services available as needed for critically ill PHO patients at your facility? (please indicate which laboratory tests are available at your facility) | *Species identification in blood, urine or CSF*  *Almost Always (24/7)*  *Frequently*  *Sometimes/Moderate availability*  *Infrequently/Limited availability*  *Almost Never*  *Not available*  *Do not know* | *Antibiotic sensitivity analyses*  *Almost Always (24/7)*  *Frequently*  *Sometimes/Moderate availability*  *Infrequently/Limited availability*  *Almost Never*  *Not available*  *Do not know* |
|  |  |  | *Fungal cultures*  *Almost Always (24/7)*  *Frequently*  *Sometimes/Moderate availability*  *Infrequently/Limited availability*  *Almost Never*  *Not available*  *Do not know* |  |
|  |  | How often is monitor of antibiotic drug levels (e.g. Vancomycin level) available as needed for critically ill PHO patients at your facility? | *Almost Always* | *Frequently* |
|  |  |  | *Sometimes/Moderate availability* | *Infrequently/Limited availability* |
|  |  |  | *Almost Never* | *Not available* |
|  |  |  | *Do not know* | |
|  |  | How often is monitoring of antineoplastic drug levels (e.g. methotrexate levels) available as needed for critically ill PHO patients at your facility? | *Almost Always* | *Frequently* |
|  |  |  | *Sometimes* | *Infrequently* |
|  |  |  | *Almost Never* | *Not available* |
|  |  |  | *Do not know* | |
|  |  | Is there a system to perform drug-dosing adjustments in critically ill pediatric patients with kidney or liver disease (e.g. computerized support alerts, prescriptions reviewed by pharmacists)? | *Yes* | |
|  |  |  | *No* | |
|  |  |  | *Do not know* | |
|  | Imaging Services | How often are Plain XR (2 views) studies available as needed for critically ill PHO patients at your facility? | *Almost Always (24/7)* | *Frequently* |
|  |  |  | *Sometimes/Moderate availability* | *Infrequently/Limited availability* |
|  |  |  | *Almost Never* | *Not available* |
|  |  |  | *Do not know* | |
|  |  | How often are Ultrasound studies available as needed for critically ill PHO patients at your facility? | *Almost Always (24/7)* | *Frequently* |
|  |  |  | *Sometimes/Moderate availability* | *Infrequently/Limited availability* |
|  |  |  | *Almost Never* | *Not available* |
|  |  |  | *Do not know* | |
|  |  | How often are Computed Tomography (CT) scans available as needed for critically ill PHO patients at your facility? | *Almost Always (24/7)* | *Frequently* |
|  |  |  | *Sometimes/Moderate availability* | *Infrequently/Limited availability* |
|  |  |  | *Almost Never* | *Not available* |
|  |  |  | *Do not know* | |
|  |  | How often are portable x-rays available to perform imaging studies at the bedside, as needed for critically ill PHO patients at your facility? | *Almost Always (24/7)* | *Frequently* |
|  |  |  | *Sometimes/Moderate availability* | *Infrequently/Limited availability* |
|  |  |  | *Almost Never* | *Not available* |
|  |  |  | *Do not know* | |
|  |  | How often are portable ultrasounds devices accessible to perform imaging studies at the bedside, as needed for critically ill PHO patients at your facility? | *Almost Always (24/7)* | *Frequently* |
|  |  |  | *Sometimes* | *Infrequently* |
|  |  |  | *Almost Never* | *Not available* |
|  |  |  | *Do not know* | |
|  |  | How often are portable echocardiogram devices accessible to perform imaging studies at the bedside, as needed for critically ill PHO patients at your facility? | *Almost Always (24/7)* | *Frequently* |
|  |  |  | *Sometimes/Moderate availability* | *Infrequently/Limited availability* |
|  |  |  | *Almost Never* | *Not available* |
|  |  |  | *Do not know* | |
|  |  | How often is a point-of-care ultrasound device available for use in critically ill PHO patients to assist with invasive procedures and augment physical examination findings (e.g., assessment for pulmonary effusion, etc.) at your facility? | *Almost Always* | *Frequently* |
|  |  |  | *Sometimes/Moderate availability* | *Infrequently/Limited availability* |
|  |  |  | *Almost Never* | *Not available* |
|  |  |  | *Do not know* | |
|  | Blood Bank/  Transfusion Services | How often are blood products available as needed for critically ill PHO patients at your facility? | *Whole Blood*  *Almost Always (24/7)*  *Frequently*  *Sometimes/Moderate availability*  *Infrequently/Limited availability*  *Almost Never*  *Not available*  *Do not know* | *Packed Red Blood Cells*  *Almost Always (24/7)*  *Frequently*  *Sometimes/Moderate availability*  *Infrequently/Limited availability*  *Almost Never*  *Not available*  *Do not know* |
|  |  |  | *Platelets*  *Almost Always (24/7)*  *Frequently*  *Sometimes/Moderate availability*  *Infrequently/Limited availability*  *Almost Never*  *Not available*  *Do not know* | *Fresh Frozen Plasma*  *Almost Always (24/7)*  *Frequently*  *Sometimes/Moderate availability*  *Infrequently/Limited availability*  *Almost Never*  *Not available*  *Do not know* |
|  |  |  | *Cryoprecipitate*  *Almost Always (24/7)*  *Frequently*  *Sometimes/Moderate availability*  *Infrequently/Limited availability*  *Almost Never*  *Not available*  *Do not know* | *Leuco-depleted blood*  *Almost Always (24/7)*  *Frequently*  *Sometimes/Moderate availability*  *Infrequently/Limited availability*  *Almost Never*  *Not available*  *Do not know* |
|  |  |  | *In general, how long does it take to obtain STAT (emergency) blood products?*  *< 1hr ii. 1-4hr iii. 4-8hrs iv. 8-12hrs v. >12hrs* | |
|  |  | How often are blood type testing (e.g., ABO and RhD grouping) and cross matching pre-transfusion available as needed for the management of critically ill PHO patients at your facility? | *Almost Always (24/7)* | *Frequently* |
|  |  |  | *Sometimes/Moderate availability* | *Infrequently/Limited availability* |
|  |  |  | *Almost Never* | *Not available* |
|  |  |  | *Do not know* | |
|  |  | How often is blood product screening for infectious diseases (e.g. HIV, Hepatitis) pre-transfusion available as needed for critically ill pediatric patients (including critically ill PHO patients) at your facility? | *Almost Always (24/7)* | *Frequently* |
|  |  |  | *Sometimes/Moderate availability* | *Infrequently/Limited availability* |
|  |  |  | *Almost Never* | *Not available* |
|  |  |  | *Do not know* | |
|  | OR and Anesthesia | How often are acute surgery services available as needed for critically ill PHO patients at your facility? | *Almost Always (24/7)* | *Frequently* |
|  |  |  | *Sometimes/Moderate availability* | *Infrequently/Limited availability* |
|  |  |  | *Almost Never* | *Not available* |
|  |  |  | *Do not know* | |
|  |  | How often is an operating room available within 30 minutes as needed for emergent surgeries in critically ill PHO patients? | *Almost Always (24/7)* | *Frequently* |
|  |  |  | *Sometimes/Moderate availability* | *Infrequently/Limited availability* |
|  |  |  | *Almost Never* | *Not available* |
|  |  |  | *Do not know* | |
| **Medication and**  **Equipment**  **Medication and**  **Equipment**  **Medication and**  **Equipment**  **Medication and**  **Equipment** | Medications | How often are first line antibiotics available for critically ill PHO patients presenting with fever and neutropenia at your facility? | *Almost Always* | *Frequently* |
|  |  |  | *Sometimes/Moderate availability* | *Infrequently/Limited availability* |
|  |  |  | *Almost Never* | *Not available* |
|  |  |  | *Do not know* | |
|  |  | How often are second or third-line antibiotics available for critically ill PHO patients with proven or suspected infection due to multi-drug resistant organisms at your facility? | *Almost Always* | *Frequently* |
|  |  |  | *Sometimes/Moderate availability* | *Infrequently/Limited availability* |
|  |  |  | *Almost Never* | *Not available* |
|  |  |  | *Do not know* | |
|  |  | How often are sedatives and analgesics (e.g. Fentanyl, Lorazepam) available when needed for critically ill PHO patients at your facility? | *Almost Always* | *Frequently* |
|  |  |  | *Sometimes/Moderate availability* | *Infrequently/Limited availability* |
|  |  |  | *Almost Never* | *Not available* |
|  |  |  | *Do not know* | |
|  |  | How often are antiemetics (e.g. Ondansetron) available when needed for management of critically ill PHO patients at your facility? | *Almost Always* | *Frequently* |
|  |  |  | *Sometimes/Moderate availability* | *Infrequently/Limited availability* |
|  |  |  | *Almost Never* | *Not available* |
|  |  |  | *Do not know* | |
|  |  | How often are anticonvulsants (e.g. Diazepam, Valproic Acid) available when needed for management for critically ill PHO patients at your facility? | *Almost Always* | *Frequently* |
|  |  |  | *Sometimes/Moderate availability* | *Infrequently/Limited availability* |
|  |  |  | *Almost Never* | *Not available* |
|  |  |  | *Do not know* | |
|  |  | How often are IV isotonic fluids (e.g. NS, LR, Hartmann's solution) available when needed for management of critically ill PHO patients at your facility? | *Almost Always* | *Frequently* |
|  |  |  | *Sometimes/Moderate availability* | *Infrequently/Limited availability* |
|  |  |  | *Almost Never* | *Not available* |
|  |  |  | *Do not know* | |
|  |  | How often are inotropes and vasoactive infusions (e.g. Dopamine, Epinephrine, Norepinephrine) available when needed for the management of critically ill PHO patients at your facility? | *Almost Always* | *Frequently* |
|  |  |  | *Sometimes/Moderate availability* | *Infrequently/Limited availability* |
|  |  |  | *Almost Never* | *Not available* |
|  |  |  | *Do not know* | |
|  |  | How often are antiarrhythmic drugs (e.g. Adenosine, Amiodarone) available when needed for critically ill PHO patients at your facility? | *Almost Always* | *Frequently* |
|  |  |  | *Sometimes/Moderate availability* | *Infrequently/Limited availability* |
|  |  |  | *Almost Never* | *Not available* |
|  |  |  | *Do not know* | |
|  |  | How often are diuretics (e.g. Furosemide) available when needed for management of critically ill PHO patients? | *Almost Always* | *Frequently* |
|  |  |  | *Sometimes* | *Infrequently* |
|  |  |  | *Almost Never* | *Not available* |
|  |  |  | *Do not know* | |
|  |  | How often are medications to treat increased intracranial pressure (e.g. Mannitol, 3% hypertonic saline) available when needed for critically ill PHO patients at your facility? | *Almost Always* | *Frequently* |
|  |  |  | *Sometimes/Moderate availability* | *Infrequently/Limited availability* |
|  |  |  | *Almost Never* | *Not available* |
|  |  |  | *Do not know* | |
|  |  | How often is parental nutrition (TPN) available when needed for critically ill PHO patients at your facility? | *Almost Always* | *Frequently* |
|  |  |  | *Sometimes/Moderate availability* | *Infrequently/Limited availability* |
|  |  |  | *Almost Never* | *Not available* |
|  |  |  | *Do not know* | |
|  | Equipment and Supplies | How often are vital sign monitoring equipment with alarms indicating critical values and continuous monitoring capabilities available at each bedside of critically ill PHO patients at your facility? | *Almost Always* | *Frequently* |
|  |  |  | *Sometimes/Moderate availability* | *Infrequently/Limited availability* |
|  |  |  | *Almost Never* | *Not available* |
|  |  |  | *Do not know* | |
|  |  | How often is pediatric-sized equipment for vital sign monitoring available, as needed for management of critically ill PHO patients at your facility? (please indicate which equipment is available at your facility) | *Non-invasive blood pressure cuffs (infant to adult size)*  *Almost Always (24/7)*  *Frequently*  *Sometimes/Moderate availability*  *Infrequently/Limited availability*  *Almost Never*  *Not available*  *Do not know* | *Pulse oximetry*  *Almost Always (24/7)*  *Frequently*  *Sometimes/Moderate availability*  *Infrequently/Limited availability*  *Almost Never*  *Not available*  *Do not know* |
|  |  |  | *EKG leads*  *Almost Always (24/7)*  *Frequently*  *Sometimes/Moderate availability*  *Infrequently/Limited availability*  *Almost Never*  *Not available*  *Do not know* | *Capnography (etCO2)*  *Almost Always (24/7)*  *Frequently*  *Sometimes/Moderate availability*  *Infrequently/Limited availability*  *Almost Never*  *Not available*  *Do not know* |
|  |  |  | *Temperature probes*  *Almost Always (24/7)*  *Frequently*  *Sometimes/Moderate availability*  *Infrequently/Limited availability*  *Almost Never*  *Not available*  *Do not know* |  |
|  |  | How often are critical supplies (crash carts, refrigerator and cabinets for medications, respiratory equipment) readily available within or immediately adjacent to the PICU (or area where critically ill PHO patients are managed) for rapid retrieval in emergencies? | *Almost Always* | *Frequently* |
|  |  |  | *Sometimes/Moderate availability* | *Infrequently/Limited availability* |
|  |  |  | *Almost Never* | *Not available* |
|  |  |  | *Do not know* |  |
|  |  | How often is pediatric-sized respiratory equipment available, as needed for management of critically ill PHO patients at your facility? (please indicate which equipment is available at your facility) | *Oral and nasal airways*  *Almost Always*  *Frequently*  *Sometimes/Moderate availability*  *Infrequently/Limited availability*  *Almost Never*  *Not available*  *Do not know* | *Oxygen and aerosol therapy masks (venturi mask, non-rebreather masks, nasal cannulas)*  *Almost Always*  *Frequently*  *Sometimes/Moderate availability*  *Infrequently/Limited availability*  *Almost Never*  *Not available*  *Do not know* |
|  |  |  | *Anesthesia masks and bags*  *Almost Always*  *Frequently*  *Sometimes/Moderate availability*  *Infrequently/Limited availability*  *Almost Never*  *Not available*  *Do not know* | *High flow nasal prongs*  *Almost Always*  *Frequently*  *Sometimes/Moderate availability*  *Infrequently/Limited availability*  *Almost Never*  *Not available*  *Do not know* |
|  |  |  | *NIV masks (CPAP and BiPAP)*  *Almost Always*  *Frequently*  *Sometimes/Moderate availability*  *Infrequently/Limited availability*  *Almost Never*  *Not available*  *Do not know* | *Endotracheal tubes (ETT) and ETT suction catheters*  *Almost Always*  *Frequently*  *Sometimes/Moderate availability*  *Infrequently/Limited availability*  *Almost Never*  *Not available*  *Do not know* |
|  |  |  | *Laryngoscope blades*  *Almost Always*  *Frequently*  *Sometimes/Moderate availability*  *Infrequently/Limited availability*  *Almost Never*  *Not available*  *Do not know* | *Ventilator circuits*  *Almost Always*  *Frequently*  *Sometimes/Moderate availability*  *Infrequently/Limited availability*  *Almost Never*  *Not available*  *Do not know* |
|  |  |  | *Laryngeal mask airways (LMAs)*  *Almost Always*  *Frequently*  *Sometimes/Moderate availability*  *Infrequently/Limited availability*  *Almost Never*  *Not available*  *Do not know* |  |
|  |  | How often are intracranial pressuring (ICP) monitors available as needed for management of critically ill PHO patients at your facility? | *Almost Always* | *Frequently* |
|  |  |  | *Sometimes/Moderate availability* | *Infrequently/Limited availability* |
|  |  |  | *Almost Never* | *Not available* |
|  |  |  | *Do not know* | |
|  |  | How often is renal replacement therapy (hemodialysis and/or peritoneal dialysis) available as needed for management of critically ill PHO patients at your facility? | *Almost Always* | *Frequently* |
|  |  |  | *Sometimes/Moderate availability* | *Infrequently/Limited availability* |
|  |  |  | *Almost Never* | *Not available* |
|  |  |  | *Do not know* | |
|  |  | How often are pediatric-sized supplies available as needed for management of critically ill PHO patients at your facility? (please indicate which supplies are available at your facility) | *Peripheral intravenous (PIV) catheters*  *Almost Always*  *Frequently*  *Sometimes/Moderate availability*  *Infrequently/Limited availability*  *Almost Never*  *Not available*  *Do not know* | *Intra-osseous (IO) needles*  *Almost Always*  *Frequently*  *Sometimes/Moderate availability*  *Infrequently/Limited availability*  *Almost Never*  *Not available*  *Do not know* |
|  |  |  | *Arterial line catheters*  *Almost Always*  *Frequently*  *Sometimes/Moderate availability*  *Infrequently/Limited availability*  *Almost Never*  *Not available*  *Do not know* | *Central line catheters*  *Almost Always*  *Frequently*  *Sometimes/Moderate availability*  *Infrequently/Limited availability*  *Almost Never*  *Not available*  *Do not know* |
|  |  |  | *Nasogastric tubes*  *Almost Always*  *Frequently*  *Sometimes/Moderate availability*  *Infrequently/Limited availability*  *Almost Never*  *Not available*  *Do not know* | *Chest tubes*  *Almost Always*  *Frequently*  *Sometimes/Moderate availability*  *Infrequently/Limited availability*  *Almost Never*  *Not available*  *Do not know* |
|  |  |  | *Urinary catheters*  *Almost Always*  *Frequently*  *Sometimes/Moderate availability*  *Infrequently/Limited availability*  *Almost Never*  *Not available*  *Do not know* | |
|  |  | Is there an equipment maintenance and servicing department at your facility? | *Yes* | |
|  |  |  | *No* | |
|  |  |  | *Do not know* | |
| **Outcomes** | Diagnosis & Outcomes | Does your facility collaborate with other regional or international centers to compare and benchmark outcomes for critically ill PHO patients? | *Yes* | |
|  |  |  | *No* | |
|  |  |  | *Do not know* | |
|  |  | Is there a system in place to report and analyze major patient adverse events occurring in critically ill PHO patients admitted in your facility? | *Yes* | |
|  |  |  | *No* | |
|  |  |  | *Do not know* | |
|  |  | How often are multidisciplinary meetings to review morbidity and mortality cases, unexpected transfers, as well as clinical incidents and near misses for critically ill PHO patients conducted at your facility? | *Monthly or more frequently* | |
|  |  |  | *Quarterly (Every 3 months)* | |
|  |  |  | *Twice a year (Every 6 months)* | |
|  |  |  | *Yearly* | |
|  |  |  | *Not available* | |
|  |  |  | *Do not know* | |
|  |  | Is there a critical care (PICU/IMCU) patient data registry that includes mortality data for hospitalized PHO/BMT patients at your facility? | *Yes* | |
|  |  |  | *No* | |
|  |  |  | *Do not know* | |

**Abbreviations**: BiPAP (Bi-level Positive Airway Pressure); BLS (Basic Life Support); BMT (Bone Marrow Transplant); CAUTI (Catheter Associated Urinary Tract Infections); CLABSI (Central Line Associated Bloodstream Infection); CPAP (Continuous Positive Airway Pressure); ICU (Intensive Care Unit); IMCU (Intermediate Medical Care Unit); LR (Lactated Ringer’s Solution); NIV (Non-Invasive Ventilation); NS (Normal Saline); PALS (Pediatric Advanced Life Support); PHO (Pediatric Hematology-Oncology); PICU (Pediatric Intensive Care Unit); VAP (Ventilator Associated Pneumonia); 24/7 (24 hours a day, 7 days a week);
